# Selective STING Activation in Intratumoral Myeloid Cells via CCR2-Directed Antibody–Drug Conjugate TAK-500

**DOI:** 10.1158/2326-6066.CIR-24-0103

**Published:** 2025-02-07

**Authors:** Vicky A. Appleman, Atsushi Matsuda, Michelle L. Ganno, Dong Mei Zhang, Emily Rosentrater, Angel E. Maldonado Lopez, Angelo Porciuncula, Tiquella Hatten, Camilla L. Christensen, Samantha A. Merrigan, Hong Myung Lee, Min Young Lee, Charlotte I. Wang, Linlin Dong, Jian Huang, Natasha Iartchouk, Jianing Wang, He Xu, Tomoki Yoneyama, Konstantin I. Piatkov, Satyajeet Haridas, Carole E. Harbison, Richard C. Gregory, Alexander Parent, Neil Lineberry, Chris Arendt, Kurt A. Schalper, Adnan O. Abu-Yousif

**Affiliations:** 1 Oncology Drug Discovery Unit, Takeda Development Center Americas, Inc. (TDCA), Lexington, Massachusetts.; 2 Department of Pathology, Yale University School of Medicine, New Haven, Connecticut.; 3 Translational Sciences, Takeda Development Center Americas, Inc. (TDCA), Lexington, Massachusetts.; 4 Drug Discovery Science, Takeda Development Center Americas, Inc. (TDCA), Lexington, Massachusetts.; 5 Oncology Cell Therapy and Therapeutic Area Unit, Takeda Development Center Americas, Inc. (TDCA), Lexington, Massachusetts.; 6 Clinical Science, Takeda Development Center Americas, Inc. (TDCA), Lexington, Massachusetts.; 7 Drug Metabolism and Pharmacokinetics, Takeda Development Center Americas, Inc. (TDCA), Lexington, Massachusetts.; 8 Global Biologics, Takeda Development Center Americas, Inc. (TDCA), Lexington, Massachusetts.; 9 OTAU/PTM, Takeda Development Center Americas, Inc. (TDCA), Lexington, Massachusetts.; 10 Ambrx, San Diego, California.; 11 Quantitative Solutions, Takeda Development Center Americas, Inc. (TDCA), Lexington, Massachusetts.; 12 R&D excellence, ZS Associates, Boston, Massachusetts.; 13 DSRE, Takeda Development Center Americas, Inc. (TDCA), Lexington, Massachusetts.; 14 Precision and Translational Medicine, Takeda Development Center Americas, Inc. (TDCA), Lexington, Massachusetts.; 15 OTAU, Takeda Development Center Americas, Inc. (TDCA), Lexington, Massachusetts.; 16 Department of Oncology, Takeda Development Center Americas, Inc. (TDCA), Lexington, Massachusetts.

## Abstract

The tumor microenvironment in solid tumors contains myeloid cells that modulate local immune activity. Stimulator of IFN gene (STING) signaling activation in these myeloid cells enhances local type-I IFN production, inducing an innate immune response that mobilizes adaptive immunity and reprograms immunosuppressive myeloid populations to drive antitumor immunity. In this study, we generated TAK-500, an immune cell–directed antibody–drug conjugate, to deliver a STING agonist to CCR2^+^ human cells and drive enhanced antitumor activity relative to nontargeted STING agonists. Preclinically, TAK-500 triggered dose-dependent innate immune activation *in vitro.* In addition, a murine TAK-500 immune cell–directed antibody–drug conjugate surrogate enhanced innate and adaptive immune responses both in *in vitro* and murine tumor models. Spatially resolved analysis of CCR2 and immune cell markers in the tumor microenvironment of >1,000 primary human tumors showed that the CCR2 protein was predominantly expressed in intratumoral myeloid cells. Collectively, these data highlight the clinical potential of delivering a STING agonist to CCR2^+^ cells.

## Teaser

The authors show that targeting a STING agonist to CCR2-expressing myeloid cells using an immune cell–directed antibody–drug conjugate increases potency and enhances antitumor activity compared with an unconjugated STING agonist.

## Introduction

Immune checkpoint inhibitors (ICI) have revolutionized cancer treatment by targeting T-cell co-inhibitory receptors such as PD-1, CTLA-4, and LAG-3 (1–4). However, only a minority of patients benefit from these agents, and those who respond often develop acquired resistance (1–3). The mechanisms mediating ICI resistance are not well established, but they include CD8^+^ T-cell dysfunction/exhaustion, tumor-cell antigen presentation defects, and the accumulation of immunosuppressive myeloid cell populations in the tumor microenvironment (TME; refs. 5–7). To address these resistance drivers, the stimulator of IFN genes (STING) pathway activation has emerged as an attractive approach to drive more potent innate and adaptive immune antitumor effects, including those that have failed or are unresponsive to immuno-oncology therapies (8).

STING is an endoplasmic reticulum signaling protein that is broadly expressed in both immune and nonimmune cells (9). STING binds 2′,3′-cGAMP produced by cyclic GMP–AMP synthase in response to cytosolic DNA and induces type-I IFNs and other proinflammatory cytokines via IFN regulatory factor-3, tank binding kinase-1, and NF-κB signaling (9–11). Type-I IFNs promote robust antiviral immunity, and accumulating evidence suggests that type-I IFNs produced by myeloid immune cells play a critical role in cancer immunosurveillance (12). In addition, productive type-I IFN–mediated antitumor immune responses require an intact STING signaling pathway within myeloid cells, including dendritic cells (DC) and macrophages, highlighting the critical role of STING signaling in innate immune cells to achieve robust and durable tumor rejection (13).

STING pathway activation has been explored as a potential anticancer treatment in human clinical trials, but with limited efficacy. A novel systemically delivered STING agonist, dazostinag, has been developed that activates innate and adaptive immune responses via type-I IFN signaling (14). However, systemic delivery of STING can activate signaling simultaneously in multiple cell types within and outside the TME, which could reduce the potency and limit the therapeutic index. We hypothesized that targeted delivery of a potent STING agonist selectively to intratumoral myeloid cells may maximize the antitumor effect of STING signaling, while minimizing undesired and potentially unproductive proinflammatory systemic responses.

Chemokine receptor-2 (CCR2) is a cell surface receptor for chemokine ligand-2 (CCL2), and its binding results in trafficking of CCR2^+^ cells along CCL2 gradients (15, 16). CCR2 can be expressed on a variety of cells but is mainly observed on leukocytes, including activated T cells and myeloid cells. During inflammation, CCL2 is upregulated in monocytes/macrophages and DCs causing these cells to mobilize from the bone marrow/blood into inflamed tissue and to differentiate into tissue-resident macrophages. In response to proinflammatory signals, these tissue-resident macrophages adopt an immunostimulatory phenotype characterized by proinflammatory cytokine production, enhanced antigen presentation, and phagocytic activity. However, they can also adopt an immunosuppressive phenotype by differentiating into myeloid-derived suppressor cells (MDSC) and establishing a tolerogenic TME (17). Many tumors present an altered microenvironment, shifting proinflammatory tumor-infiltrating myeloid cells to protumor phenotypes that hinder productive antitumor immune responses and tumor elimination (17, 18).

In this study, we show that TAK-500, an immune cell–directed antibody–drug conjugate (iADC) that selectively binds to and activates STING in CCR2-expressing human cells, enhances local type-I IFN production and generates adaptive immune responses while potentially minimizing the undesired and dose-limiting proinflammatory systemic responses of nontargeted STING agonists. Preclinical studies using mTAK-500, a murine cross-reactive surrogate of TAK-500, demonstrated antitumor activity both as monotherapy and in combination with anti–PD-1 and local irradiation. The antitumor activity of the CCR2-directed iADC was associated with enhanced activation of both innate and adaptive immune responses, supporting the immunostimulatory impact of the treatment.

Using multiplex quantitative immunofluorescence (mQIF) analysis, we identified CCR2 protein expression in intratumoral myeloid cells from multiple human solid tumor cohorts, with prominent association with local adaptive immune responses and survival, supporting a translational strategy for targeting CCR2 with a STING agonist via an iADC strategy in human malignancies.

## Materials and Methods

### Cell lines and mouse models

All animal experiments were performed in compliance with and approval from the Takeda Institutional Animal Care and Use Committee. All cell lines used in the experiments were obtained from the indicated sources summarized below, cultured in appropriate media as summarized below, expanded, and then banked into the Takeda Oncology cell bank as high-density frozen vials stored in liquid nitrogen between passages 3 and 6. For cell line reporter assays, cells were thawed from high-density frozen vials and then used immediately for experiments after a short recovery period. Cells for *in vivo* implantation were thawed from frozen vials and cultured a short time before use in the experiment. During the culture time, cells were fed and split as needed to avoid over-confluence. Cells were used for experiments as needed during the period of culture but never exceeding passage 10. Cells were *Mycoplasma* and murine pathogen tested via the IMPACT I assay by IDEXX BioAnalytics (the most recent testing dates are indicated in Supplementary Table S1). CT26.WT and MC38 cell lines were additionally verified using IDEXX CellCheck Cell Line Authentication Service with 27-Marker STR Strain Analysis.

Individual cell line details: THP1-Dual human acute myeloid leukemia (AML) cells were obtained from InvivoGen (Cat. #thpd-nfis) and cultured in RPMI-1640 (Gibco, Cat. #11875093) with 2 mmol/L L-glutamine (Gibco, Cat. #A2916801), 25 mmol/L 4-(2-hydroxyethyl)-1-piperazine-1-ethane-sulfonic acid (HEPES, Gibco, Cat. #15630130), 10% heat-inactivated FBS (HI-FBS, Gibco, Cat. #A5670501), and 100 µg/mL Normocin (InvivoGen, Cat. #ant-nr-05) and with 100 units (U)/mL–100 μg/mL Penicillin–Streptomycin (Gibco, Cat. #15070063), 10 μg/mL blasticidin (Gibco, Cat. #A1113902), 1 μg/mL puromycin (Gibco, Cat. #A1113802), and 100 μg/mL Zeocin (Gibco, Cat. #R25005) and last *Mycoplasma* tested on June 19, 2017. CT26.WT cells were obtained from ATCC (Cat. #CRL-2638), cultured in RPMI-1640 with 10% FBS (Gibco, Cat. #A5670701), and last *Mycoplasma* tested on June 23, 2016. MC38 cells were obtained from James W. Hodge at the NCI, cultured in DMEM (Gibco, Cat. #11966025) with 10% FBS, and last *Mycoplasma* tested on July 10, 2020. The THP1-Dual KI-R232 cells were obtained from InvivoGen (Cat. #thpd-r232). This cell line was derived at InvivoGen from the human THP1 (AML) cells by stable integration of the Lucia luciferase gene, a secreted luciferase reporter gene, under the control of an ISG54 minimal promoter in conjunction with five IFN-stimulated response elements. These cells were cultured in RPMI-1640 with 2 mmol/L L-glutamine, 25 mmol/L HEPES, 10% HI-FBS, and 100 µg/mL Normocin and 100 units of Penicillin–Streptomycin.

### TAK-500 synthesis

Tris(2-carboxyethyl) phosphine (1 mmol/L solution in water, 2.5 equivalents, Thermo Fisher Scientific, Cat. #T2556) was added to a solution of anti-CCR2 (TAK-202, 10 mg/mL, generated as described in US 7473421 B2) in 50 mmol/L histidine (Thermo Fisher Scientific, Cat. #A10413.36) and 125 mmol/L arginine (Thermo Fisher Scientific, Cat. #A15738.36), pH 6.1 buffer. The reaction mixture was purged with argon and incubated at room temperature (RT) for 3 hours with gentle shaking. The linker–payload construct [5 mmol/L solution in dimethylacetamide (Thermo Fisher Scientific, Cat. #115690250), 7 equivalents] was then added slowly into the above mixture. The reaction was purged with argon and incubated at RT for another 1 hour with gentle shaking. The reaction mixture was purified on a Gilson Preparative high-performance liquid chromatography (HPLC) system with UV detector using a size-exclusion chromatography (SEC) column (GE Superdex 200 Increase 10/300 GL, Cat. #28-9909-44, 50 mmol/L histidine, 125 mmol/L arginine, pH 6.1 buffer, 1 mL/minute). The ADC concentration, percentage aggregation, and drug–antibody ratio (DAR) were determined by UV absorbance measured by NanoDrop 2000c, analytical SEC, and analytical hydrophobic interaction chromatography (HIC)/liquid chromatography–quadruple time of flight tandem mass spectrometry (LC-QTOF), respectively. The average DAR was 3.5 to 4.5.

The linker–payload synthesis has been reported in patent WO2020229982.

### mTAK-500 synthesis

To a solution of anti-mCCR2 (RtMuMC-21-S98), 3.4 mg/mL, as described previously (19) in 50 mmol/L histidine, 125 mmol/L arginine, pH 6.1 buffer, was added 0.5 mol/L Tris (Thermo Fisher Scientific, Cat. #17926), 25 mmol/L EDTA (Thermo Fisher Scientific, Cat. #R1021), pH 8 buffer to adjust the pH to 7. Tris (2-carboxyethyl) phosphine (10 mmol/L solution in water, 20 equivalents) was then added. The reaction mixture was purged with argon and incubated at 37°C for 1.5 hours with gentle shaking. The resulting mixture was purified on a Gilson Preparative HPLC system with UV detector using a SEC column (GE Superdex 200 Increase 10/300 GL, 50 mmol/L histidine, 125 mmol/L arginine, pH 6.1 buffer, 1 mL/minute) and concentrated to ∼5 mg/mL. Dehydroascorbic acid [(Thermo Fisher Scientific, Cat. #250932500, 2 mmol/L in DMSO, Thermo Fisher Scientific, Cat. #J66650.AK), 2.2 equivalents] at 4°C was added to the purified antibody, and the resulting mixture was kept at 4°C overnight. The reaction was warmed to RT, and then the linker–payload construct (5 mmol/L solution in dimethylacetamide, 5 equivalents) was added slowly. The reaction was purged with argon and incubated at RT for 2 hours with gentle shaking. The resulting mixture was purified on a Gilson Preparative HPLC system with UV detector using a SEC column (GE Superdex 200 Increase 10/300 GL, 50 mmol/L histidine, 125 mmol/L arginine, pH 6.1 buffer, 1 mL/minute). The ADC concentration, percentage aggregation, and DAR were determined by UV absorbance measured by NanoDrop 2000c, analytical SEC, and analytical HIC/LC-QTOF, respectively. The average DAR was 2.5 to 4.0.

For *in vivo* efficacy experiments, KTI-mIgG2a-LAGA-TAK-676 was used as the isotype control for mTAK-500.

### Analytic methods for assessing TAK-500 and mTAK-500 synthesis quality

Spectra can be found in Supplementary Fig. S1 (TAK-500) and Supplementary Fig. S2 (mTAK-500). SEC spectra were recorded on a Hewlett-Packard HP1100 or an Agilent 1100 Series LC system with diode array detector using an SEC column (typically Tosoh Biosep TSK-Gel, G3000SWXL; P/N 8541; 250 Å; 5 µm; 7.8 mm × 300 mm) at 280 nm. Mobile phase was 100 mmol/L sodium phosphate (Thermo Fisher Scientific, Cat. #447982500), 300 mmol/L sodium chloride (Thermo Fisher Scientific, Cat. #447302500), pH 6.8, 10% acetonitrile (ACN, Thermo Fisher Scientific, Cat. #047138.M1; v/v) or 1× PBS (Gibco, Cat. #10010-023). A typical run was isocratic at a flow rate of 1 mL/minute for 20 minutes.

HIC spectra were recorded on a Hewlett Packard HP1100 or Agilent 1100 Series LC system with diode array detector using an HIC column (typically Tosoh Butyl-NPR, 4.6 × 35 mm, 2.5 µm, P/N: 14947) at 280 nm. Mobile phase A was 25 mmol/L sodium phosphate (Thermo Fisher Scientific, Cat. #447982500), 1.5 mol/L ammonium sulfate (Thermo Fisher Scientific, Cat. #J64419.A3), pH 7, and mobile phase B was 75% 25 mmol/L sodium phosphate, pH 7, 25% isopropanol (Thermo Fisher Scientific, Cat. #383910025). For a typical 20-minute run, a 12-minute linear gradient from 95%/5% A/B to 100% B would be used between the initial and final intervals of isocratic flow.

LC-QTOF/MS (LC/MS) spectra were recorded on an Agilent 1260 Bio-Inert Series LC system connected to an Agilent 6545 QTOF MS using a reverse-phase column heated to 80°C (typically Agilent, PLRP-S, 5 µm, 1,000 Å, 2.1 × 50 mm). Various gradients and run times were selected to best characterize the compounds. Mobile phases were based on ACN/water gradients and contained 0.1% formic acid (Thermo Fisher Scientific, Cat. #270480250). One example of a solvent gradient that was used was 95% mobile phase A (mobile phase A = 99% water + 1% ACN + 0.1% formic acid) to 100% mobile phase B (mobile phase B = 95% ACN + 5% water + 0.1% formic acid) with conditions shown in Supplementary Table S2.

Samples were either intact or reduced (20 µL of 1–5 mg/mL ADC solution treated with 4 µL of 0.5 mol/L 1,4-dithiothreitol (Thermo Fisher Scientific, Cat. #20290) solution at 37°C for 30 minutes). Raw data were deconvoluted within appropriate mass range using Agilent BioConfirm software to obtain protein molecular weights, and the Agilent DAR Calculator was used to calculate DAR.

### Assessment of mTAK-500–induced STING activation *in vitro*

The THP1-Dual KI-hSTING-R232 cells were generated at InvivoGen from the human THP1 AML cells by stable integration of the Lucia luciferase gene, a secreted luciferase reporter gene, under the control of an ISG54 minimal promoter in conjunction with five IFN-stimulated response elements. On the day of the experiment, the cells were plated to white 384-well plates (Corning, Inc., Cat. #164610) at 10,000 cells/25 μL per well density in growth media. The cell plates were dosed with 5 μL of the testing samples using the automated Bravo Liquid Handling Platform (Agilent Technologies). The dose ranges were as follows: for the positive control dazostinag (14), 10 points of threefold serial dilution from 25 µmol/L top concentration; for the test articles TAK-500 and mTAK-500, 10 points of threefold serial dilution from 500 nmol/L top concentration. After dosing, the cell plates were incubated at 37°C, in 5% CO_2_ for 20 hours. At the end of the incubation, 10 μL/well of the QUANTI-Luc (InvivoGen, ID rep-qlc1) was added, and luminescence was measured immediately using the LEADSeeker imaging system (GE HealthCare Life Sciences).

### Assessment of mTAK-500 receptor occupancy in murine whole blood

For murine receptor occupancy (RO), whole blood samples from five BALB/c mice were collected at Charles River Laboratories Ashland. A minimum of 1 mL of murine whole blood was collected from each mouse into EDTA tubes (Becton Dickinson, Cat. #367835). *In vitro* RO of mTAK-500 in murine whole blood samples was determined by measuring bound fraction of mTAK-500 to its target, CCR2, using flow cytometry on cell subsets in the whole blood. Each murine blood sample [50 μL/well, plated in a 2.2 mL/well 96-well master block plate (Thermo Fisher Scientific, Inc., Cat. #AB0661)] was treated separately with mTAK-500 (10 μL) at concentrations of 0.0005, 0.002, 0.008, 0.032, 0.128, 0.512, 2.048, 8.192, 32.77, 131.1, and 175 μg/mL (ADC) or 1× PBS as the negative control for 60 minutes at 4°C. After 60 minutes of incubation, 1,000 μL of VersaLyse Lysing solution (Beckman Coulter, Inc., Cat. #A09777) was added to each sample and mixed by vortexing. The mixed samples were incubated at RT for approximately 20 minutes in the dark. After incubation, the 96-well plate was centrifuged at 400*g* for approximately 5 minutes at 4°C with the brake on. The supernatant of each well was aspirated, and the plate was vortexed for approximately 10 seconds. The 1× PBS (1,800 μL) was pipetted into each well of 2.2 mL/well plate containing sample and mixed by pipetting up and down at least three times for mixing.

Cells were resuspended with 100 μL per each well of Live/Dead stain working solution [Live/Dead Fixable Near-IR Dead Cell Stain solution (Thermo Fisher Scientific, Inc., Cat. #L10119, diluted 1:1,000 in 1× PBS)], and the plate was vortexed for approximately 10 seconds. The samples in the plate were incubated for a minimum of 30 minutes in a refrigerator set to maintain 4°C, protected from light. After incubation, 1,800 µL of the stain buffer (BD Biosciences, Cat. #554656) was added to each well of the 96-well plate and mixed by pipetting up and down at least three times. The 96-well plate was centrifuged at approximately 400*g* for approximately 5 minutes at approximately 4°C with the brake on; the supernatant was aspirated and the 96-well plate was vortexed for approximately 10 seconds to resuspend the cell pellet. Ten microliters of Blocking Buffer Working Solution (Fc Block diluted 1:50 in stain buffer) was pipetted into each well of the plate and vortexed for approximately 10 seconds. The samples in the plate were incubated for approximately 10 minutes on wet ice in the dark.

To detect mTAK-500 binding to CCR2, cells were resuspended in 50 μL of either PE staining cocktail (full stained samples) containing detection antibody IgG2a-PE (rat anti–mouse IgG2a secondary antibody, PE from Thermo Fisher Scientific, Inc., Cat. #12-4210-82) or stain buffer only [fluorescence minus one (FMO) and setup control wells]. Fifty microliters of stain buffer containing the following antibodies: CD3-BV510, B220-BV510, NK1.1-BV510, CD11b-PerCP-Cy5.5, CD43-PE-Cy7, CD11c-APC, and Ly6C Super Bright 436 was then added to each well except for the positive setup control (See Supplementary Table S3 for staining cocktail details and Supplementary Fig. S3 for gating strategy). CD192-PE was added to the positive setup control, which was used as the positive control for staining in this study. After incubation for a minimum of 20 to 30 minutes at 4°C, stain buffer (1,800 μL) was pipetted into each well, and the plate was centrifuged at approximately 400*g* for approximately 5 minutes at 4°C. The supernatant was aspirated, and the plate was vortexed for approximately 10 seconds to resuspend the cell pellet. Two hundred microliters of stain buffer was pipetted into each well of the plate and mixed by pipetting up and down at least three times. The samples were pipette-transferred from the plate to the corresponding wells of a Corning 96-well V-shaped plate (Corning, Cat. #CLS3894-50EA). Samples were analyzed using a BD FACSCanto II Flow Cytometer (BD Biosciences), with a target of 30,000 live cell events for each antibody to be collected as the stopping gate, and data were recorded using BD FACSDiva software, version 8.0.1 (BD Biosciences).

### Assessment of TAK-500 receptor occupancy in human whole blood

After written consent was obtained and under an approved Institutional Review Board (IRB) protocol at an FDA-registered collection center, blood samples from five healthy human donors were collected at Charles River Laboratories Mattawan and Charles River Laboratories Ashland sites, respectively. Patients were screened for hepatitis B virus (HBV), hepatitis C virus (HCV), human immunodeficiency virus (HIV), human T-cell lymphotrophic virus (HTLV), West Nile virus (WNV), *Trypanosoma cruzi*, and syphilis prior to collection and excluded for positive results. A minimum of 10 mL of human whole blood was collected from each donor in sodium heparin (158-USP-unit) BD Vacutainer blood collection tubes (BD Biosciences, Cat. #367874). Lymphocyte count (absolute) and monocyte count (absolute) were evaluated from each human and nonhuman primate (NHP) blood sample using the ADVIA 120 (Siemens Healthineers). *In vitro* RO of TAK-500 in human whole blood samples was determined by measuring bound fraction of TAK-500 to its target, CCR2, using flow cytometry on cell subsets in the whole blood. Each blood sample (90 μL/well, plated in a 96-well master block plate) was treated separately with TAK-500 (10 μL) at concentrations of 0.25, 0.5, 1, 2, 4, 8, 16, 32, 64, 128 μg/mL (by ADC concentration) or 1× PBS as the negative control for 60 minutes at 4°C. After 60 minutes of incubation, 1,800 μL of 1× red blood cell (RBC) lysis solution (Thermo Fisher Scientific, Cat. #00-4300-54) was added to each sample and mixed by pipetting. The mixed samples were incubated at RT for approximately 15 to 20 minutes in the dark. After incubation, the 96-well plate was centrifuged at 400*g* for approximately 5 minutes at 4°C with the brake on. The supernatant of each well was aspirated, and remaining cell pellets were ready for Live/Dead staining.

Cell pellets were resuspended with 100 μL per each well of Live/Dead stain working solution [Live/Dead Fixable Near-IR Dead Cell Stain solution (Thermo Fisher Scientific, Cat. #L10119) diluted 1:1,000 in 1× PBS], and the plate was vortexed for approximately 10 seconds. The samples in the plate were incubated for a minimum of 30 minutes in a refrigerator that was set to maintain 4°C protected from light. After incubation, the stain buffer (1,800 μL) was added to each well of the 96-well plate and mixed by pipetting up and down at least three times. The 96-well plate was centrifuged at approximately 400*g* for approximately 5 minutes at approximately 4°C with the brake on, and the supernatant was aspirated. Ten microliters of Blocking Buffer Working Solution (Fc Block diluted 1:50 in stain buffer) was pipetted into each well of the plate and vortexed for approximately 10 seconds. The samples in the plate were incubated for 10 ± 2 minutes at 4°C, protected from light.

To detect TAK-500 binding to CCR2, the cells were resuspended in 50 μL of either PE staining cocktail (full stained samples) containing detection antibody IgG-PE or stain buffer only (FMO; and setup control). After incubation for a minimum of 20 to 30 minutes at 4°C protected from light. Stain buffer (1,800 μL) was pipetted into each well, and the plate was centrifuged at approximately 400*g* for approximately 5 minutes; the supernatant was aspirated; and the plate was vortexed for approximately 10 seconds to resuspend cell pellet.

Fifty microliters of stain buffer containing the following antibodies was added to the samples (except for the positive setup control): HLA-DR-FITC, CD3-PE-Cy7, CD20-PE-Cy7, CD56-PE-Cy7, CD16-APC, and CD14-BV510 (See Supplementary Table S4 for details). Fifty microliters of stain buffer containing the following antibodies was added to the setup control: HLA-DR-FITC, CD3-PE-Cy7, CD20-PE-Cy7, CD56-PE-Cy7, CD16-APC, CD14-BV510, and CD192 (CCR2)-PE (See Supplementary Table S4 for details). Setup control was used as the positive control for staining in this study. The samples in the plate were incubated for a minimum of 20 to 30 minutes at 4°C protected from light. After incubation, the stain buffer (1,800 μL) was pipetted into each well, and samples were mixed by pipetting up and down at least three times. The plate was centrifuged at approximately 400*g* for approximately 5 minutes at 4°C, the supernatant was aspirated. Cells were resuspended in 500 μL of stain buffer, and 300 μL of each sample was pipette-transferred to a 96-well V-bottom plate (MilliporeSigma).

Samples were analyzed with a BD FACSCanto II Flow Cytometry with a target of 60,000 lymphocytes/monocytes cell events to be collected as the stopping gate, and data were recorded using the BD FACSDiva software (version 8.0.1); see Supplementary Fig. S4 for gating strategy.

### *In vitro* assessment of monocyte activation in human peripheral blood mononuclear cells

Blood was collected from five healthy donors at STEMCELL Technologies using IRB-approved consent forms and protocols. Donors were tested and found to be negative for HIV-1 and HIV-2, hepatitis B, and hepatitis C prior to donation. Blood from each donor was diluted separately into 50-mL conical tubes to include 25 mL of blood and 10 mL of PBS plus 2% HI-FBS (10 mL HI-FBS plus 500 mL PBS) in each tube. Blood and PBS were then mixed by pipetting up and down with a 10-mL pipette several times. Thirteen milliliters of Ficoll was added (Lymphoprep: STEMCELL Technologies No. 07851; Lot 00119) with a 10-mL pipette by carefully and slowly pipetting the Ficoll at the bottom of each blood tube to underlay the Ficoll. Tubes were then centrifuged at RT at 800*g* for 20 minutes with no brake and acceleration set to 5. As the centrifuge slowed down below 40 *g*, the acceleration and deceleration were then increased to nine and nine, respectively, and left at those settings until the end of the spin. The tubes then had plasma/serum at the top, peripheral blood mononuclear cells (PBMC; buffy coat) as the middle layer, and Ficoll plus RBCs and granulocytes as the bottom layer. The top layer was slowly aspirated down to 25 mL, leaving 5 to 7 mL of plasma/serum above the buffy coat. The buffy coat was then carefully removed using a 5-mL pipette, avoiding the bottom layer. The volume of all tubes was then adjusted to 50 mL with PBS plus HI-FBS, and the samples were mixed by inverting the tubes. The tubes were centrifuged at 400*g* for 10 minutes at RT with the brake on. The supernatant was aspirated by tilting the tube while being careful to avoid the cell pellet. Pellets were resuspended in 10 mL of PBS plus HI-FBS. Samples were then centrifuged at 400*g* for 10 minutes at RT, and the supernatant was removed. The pellets were resuspended in 10 mL of ACK Lysing buffer, ensuring that there were no clumps. The tubes were transferred to ice for 5 minutes, and then the samples were diluted with PBS plus HI-FBS to a total volume of 50 mL. Samples were centrifuged at 400*g* for 10 minutes at RT. The pellet was checked to ensure that it was no longer pink. The pellets were then resuspended in a total volume of 50 mL PBS plus HI-FBS buffer, and PBMCs were kept on ice until ready for use.

Human PBMCs were treated in triplicate with TAK-500 at final concentrations of 1,000, 200, 40, 8, 1.6, 0.32, 0.064, 0.0128, and 0.00256 nmol/L by dazostinag payload. Each donor was also treated with PBS vehicle control in triplicate. For treatment, 20 μL of the appropriate treatment solution was added to each well containing 180 μL of RPMI plus 10% FBS plus human PBMCs. The plate was then incubated for 24 hours ± 1 hour in a humidified incubator set to maintain 37°C and 5% CO_2_.

After the incubation with TAK-500 or vehicle, plates were centrifuged for 5 minutes at 500 relative centrifugal force (RCF) in an Allegra X-14R centrifuge (Beckman Coulter Inc.) set at 4°C. The supernatant was removed, and the cells were washed one time with 1× PBS. The plates were centrifuged again for 5 minutes at 500 RCF in an Allegra X-14R centrifuge (Beckman Coulter Inc.) set at 4°C. The supernatant was removed, and the cells were resuspended in Accutase for 15 minutes at 37°C. The plates were then centrifuged again for 5 minutes at 500 RCF in an Allegra X-14R centrifuge set at 4°C. The supernatant was removed, and the cells were washed in cold staining buffer. After the wash, cells were resuspended in cold staining buffer and stained with the antibody panel listed in Supplementary Table S5.

The 96-well U-bottom plates with single-cell suspension of all samples was centrifuged for 5 minutes at 500 RCF in an Allegra X-14R centrifuge set at 4°C. The supernatant was removed and washed one time with 1× PBS. The supernatant was then removed for cell surface staining. The cells were resuspended in 100 μL of Live/Dead Fixable Dead Cell Stain (Invitrogen) at a 1:1,000 dilution in 1× PBS without calcium and magnesium. The cells were incubated at RT for 10 minutes and protected from light. After incubation, cells were washed one time by adding 200 μL of 1× PBS to each well and centrifuged for 4 minutes at 500 RCF in an Allegra X-14R centrifuge set at 4°C. After centrifuging, the supernatant was discarded, and the cells were washed with 200 μL of 1× PBS twice more.

The cells were resuspended in 50 μL of Fc blocking solution containing 20 μg/mL Fc Block (Bio X Cell, Cat. #BE0307) in 1× PBS plus 2% HI-FBS (which will be referred to as “staining buffer” for the remainder of the protocol). Fc Block is compatible with the CD16 antibody in the surface stain, as they do not recognize the same epitopes on CD16. The plates were incubated at approximately 4°C for 10 minutes and protected from light. After this incubation, 50 μL of a 2× surface stain mixture (comprising the listed antibodies in Supplementary Table S5, diluted in staining buffer at twice the indicated concentrations) was added to each well. The cells were incubated at 4°C for 30 minutes and protected from light. Cells were then pelleted for 4 minutes at 500 RCF in an Allegra X-14R centrifuge set at 4°C and washed twice with stain buffer.

Cells were next resuspended in 200 μL of 1× Fixation/Permeabilization Working Solution from the eBioscience Foxp3/Transcription Factor Staining Buffer Set (Invitrogen, Cat. #00-5523-00) and incubated at RT for 30 minutes while being protected from light. Cells were then pelleted for 5 minutes at 400 RCF in an Allegra X-14R centrifuge set at 4°C and washed with 200 μL of staining buffer. The wash was repeated twice. Cells were then stored in 200 μL of staining buffer until ready for data acquisition. Data acquisition for each sample was performed no later than 4 days after staining was complete and was done using an LSRFortessa (Becton, Dickinson and Company); see Supplementary Fig. S5 for gating strategy.

### CRISPR knockout and functional assessment of CCR2 in THP1 cells

THP1-Dual cells were elecroporated with CRISPR ribonuceloproteins loaded with human CCR2 synthetic single-guide RNA guides (included in CCR2 Gene Knockout Kit v2, Synthego) according to the manufacturer’s protocol. The sequences for the single-guide RNA guides were GAU​AAA​CCG​AGA​ACG​AGA​UG, ACC​UUU​UUU​GAU​UAU​GAU​UA, and GUA​GAG​CGG​AGG​CAG​GAG​UU. After electroporation, the cells were expanded in culture and profiled for CCR2 expression as a means of characterizing editing efficiency by flow cytometry using anti-human CCR2 (BioLegend, Cat. #357234). The cells that were edited to be CCR2 negative were further purified by negative selection from the edited pool by anti-biotin MACS (Miltenyi Biotec, Cat. #130-090-485), after a 30-minute incubation with biotinylated anti-human CCR2 antibody (5 µg/mL, BioLegend, Cat. #357234). Purified CCR2 KO cells were then expanded in culture to obtain adequate cell numbers prior to function assays.

To assess the impact of CCR2 knockout on functional readouts, THP1-Dual WT or THP1-Dual CCR2 KO cells were plated at 1 × 10^5^ cells per well in TC-treated flat-bottom plates. Ten-fold serial dilutions of TAK-500, mTAK-500, KTI-TAK-676, or mKTI-TAK-676 were prepared at twice the final concentrations in a separate 96-well polystyrene plate. Calculated drug concentrations were normalized to DAR of each ADC. Drug titrations were added to cells, and plates were incubated overnight. After overnight incubation, 15 µL of cell supernatants was transferred to a white flat-bottom 96-well plate containing 50 µL of reconstituted QUANTI-Luc (InvivoGen, Cat. #rep-qlc1) and immediately read on a luminometer.

### *In vitro* assessment of cytokine induction in human whole blood

Blood was collected from nine healthy donors at STEMCELL Technologies using IRB-approved consent forms and protocols. Donors were tested and found to be negative for HIV-1 and HIV-2, hepatitis B, and hepatitis C prior to donation. Blood was collected in sodium heparin Vacutainer tubes and rocked for homogenization. After removing the rubber caps, 270 μL of whole blood was aliquoted into each well, at 20 wells/donor, of U-bottom tissue culture plates at RT. Immediately after dispensing blood, 30 μL of the respective treatment solution was added to each well, and samples were mixed by gently pipetting 30 μL up and down for five times. The same procedure was repeated for each donor using fresh tips. One well per sample per donor was treated. Plates were then placed at 37°C in 5% CO_2_ humidified incubator for 24 ± 1 hour.

For plasma collection, plates containing 300 μL of treated whole blood were centrifuged at 10,000 rpm for 10 minutes in a Sorvall Legend XFR centrifuge set at 4°C. Approximately 80 to 120 μL of plasma was then transferred into U-bottom ultralow attachment plates (Thermo Fisher Scientific, Cat. #174927). Plasma samples were again centrifuged at 10,000 rpm for 10 minutes to further clarify the plasma and remove any leftover cells that may have been transferred. This centrifugation for clarification was repeated once more, and after this last transfer, the plates were sealed with a plastic film, snap-frozen on dry ice, and stored frozen at less than −70°C.

Plasma samples were thawed on ice and centrifuged for 10 minutes at 10,000 rpm in an Sorvall Legend XFR centrifuge at 4°C to pellet any aggregates in the samples. The supernatant was then collected for further processing.

To be able to assess cytokine concentration, plasma supernatant samples were diluted 16-fold for this assay to evaluate eotaxin, eotaxin-3, IFN-γ, IFN-α2a, IFN-β, IL-10, IL-12p70, IL-13, IL-1β, IL-2, IL-29/IFN-λ1, IL-4, IL-6, IL-8, monocyte chemoattractant protein-1 (MCP-1), MCP-4, macrophage-derived chemokine, macrophage inflammatory protein-1α, thymus and activation-regulated chemokine, and TNF-α. Plasma supernatant samples were diluted 32-fold to evaluate the level of IFN-gamma inducible protein 10 (IP-10) and macrophage inflammatory protein-1β. Specimens were assessed as single samples.

The concentrations of all cytokines were measured using a combination of three kits from Meso Scale Discovery (Cat. #K15094K-2, K15047D-2, and K15049G-2) according to the user manual. Briefly, for U-PLEX kit, provided capture antibodies were conjugated to linkers at RT, followed by an incubation on Sector plates in order to bind them to specific spots within the plates. V-PLEX plates were provided with this step already performed and validated by manufacturer. After washing all plates, plasma samples were diluted. Plates were then incubated with a detection antibody using a Sulfo-Tag (Meso Scale Diagnostics, LLC). A substrate-containing reading buffer was added, and the plates were read using the Meso Scale Discovery (Meso Scale Diagnostics, LLC) platform, an electroluminescence-based instrument, with Methodical Mind software, version 6.1.1 (Meso Scale Diagnostics, LLC).

### Evaluation of CCR2 expression levels in human dissociated tumor cells

Breast cancer–dissociated tumor cells were obtained from 23 patients via Discovery Life Sciences. Samples were collected and banked by Discovery Life Sciences after surgical resection and had been obtained using an IRB-approved and consented process. Samples were thawed at 37°C and washed with RPMI + 10% FCS. Samples were then incubated at RT for 15 minutes with DNase (Thermo Fisher Scientific, Cat. #89836), filtered in a 100 µmol/L cell strainer, and plated in a 96-well plate V-bottom plate. Cells were stained with live dead markers for 15 minutes at RT, followed by treatment with Fc Block for 20 minutes at 4°C. Cells were then incubated with extracellular staining cocktail (see Supplementary Tables S6 and S7) for 20 minutes at 4°C. After extracellular staining, cells were washed with staining buffer (BD Biosciences, Cat. #554657) and incubated with 1× Fix/Perm Buffer (Thermo Fisher Scientific, Cat. #GAS004) for 30 minutes at 4°C. Cells were then washed in 1× Fix/Perm Buffer and incubated with intracellular staining cocktail (See Supplementary Tables S6 and S7) at RT for 45 minutes. Cells were then washed in staining buffer, resuspended in staining buffer, and analyzed on a BD Fortessa. See Supplementary Figs. S6 and S7 for gating strategy.

### Evaluation of TAK-500 treatment on T-cell and NK-cell activation

Blood was collected from five healthy donors via the Millenium volunteer blood draw program using IRB-approved consent forms and protocols. One hundred thirty-five microliters of human whole blood from each of the five healthy donors was pipetted separately into each well of five 96-well round-bottom plates. Immediately after pipetting, the blood samples were treated with 0.3, 1, 3, 10, 30, 100, 300, or 1× PBS as the negative control. Treated whole blood was incubated for 24 hours in a humidified 37°C, 5% CO_2_ incubator.

An aliquot of each treated whole blood sample (100 μL) was pipetted into a deep-well 96-well V-bottom plate, and 1.8 mL of 2× RBC lysis buffer (BD Biosciences, Cat. #555899) was added to each well. The mixtures in each well were mixed up and down at least 10 times with a multichannel pipette. The plates were sealed with an aluminum plate sealer and incubated at approximately 37°C for approximately 15 minutes and protected from light. After incubation, the deep-well plates were centrifuged for approximately 5 minutes at 500*g* at RT. The plate seal was removed, and the supernatant was decanted without disturbing the cell pellets. The cell pellets were gently resuspended with 2 mL stain buffer (BD Biosciences, Cat. #554657) and centrifuged again for approximately 5 minutes at 500*g* at RT. After centrifugation, the supernatant was carefully decanted. One millliliter of 2× RBC lysis buffer was added to each well of the deep-well plates, and the lysis was repeated. After decanting the supernatant, cells were ready for staining.

To stain cell surface markers, cells were resuspended in 127 μL of antibody mixture (see Supplementary Table S8 for antibody details). Cells were transferred to a 96-well V-bottom plate and were incubated for approximately 25 minutes at approximately 4°C and protected from light. After incubation, the plates were centrifuged for approximately 5 minutes at 500*g* at RT. The supernatant was decanted without disturbing the cell pellets. The cells were resuspended in 200 μL of stain buffer to remove excess antibodies. The plates were centrifuged for approximately 5 minutes at 500*g* at RT. The supernatant was decanted without disturbing the cell pellets. The cell pellets were resuspended in 200 μL of stain buffer containing Live/Dead stain and incubated at 4°C for 10 minutes. The data were collected by using BD FACSCelesta Flow Cytometer (see Supplementary Fig. S8 for gating strategies).

### Assessment of mTAK-500 pharmacokinetics in tumor-bearing mice

Female C57BL/6 mice (The Jackson Laboratory, Cat. #000664), aged 7 to 8 weeks, were inoculated subcutaneously with 1.0 × 10 MC38 tumor cells in the right flank. When tumors grew to approximately 300 to 500 mm^3^, the animals were assigned into groups (*n* = 3/timepoint). Each group of animals received a single mTAK-500 administration via intravenous dosing at 2, 10, and 50 µg/kg dose levels by payload. Animals were sacrificed at defined timepoints, and tumor and plasma samples were harvested according to approved research operating procedures.

For plasma collection, approximately 500 μL of whole blood was obtained via cardiac puncture, placed into tubes coated with dipotassium-EDTA (K_2_EDTA) to prevent clotting, and centrifuged at 10,000 rpm for 5 minutes. Approximately 200 μL of plasma was then transferred into 1.4-mL sterile tubes, snap-frozen on dry ice, and stored frozen at approximately −80°C. Tumor samples were excised from the mice and placed into 1.4-mL sterile tubes, snap-frozen on dry ice, and stored frozen at approximately −80°C for pharmacokinetics (PK) analysis. Samples were shipped on dry ice to Frontage Laboratories, Inc., where they were stored in a freezer set to maintain −70°C ± 10°C until analysis.

The analysis of plasma and tumor samples was performed by personnel at Frontage Laboratories, Inc. following qualified LC/MS-MS methods: BTM-3181-R0 for total antibody and conjugated dazostinag from mTAK-500 in mouse K_2_EDTA plasma; BTM-3180-R0 for deconjugated dazostinag mouse K_2_EDTA plasma; BTM-3183-R0 for total antibody and conjugated dazostinag from mTAK-500 in mouse tumor homogenate; and BTM-3182-R0 for deconjugated dazostinag in mouse tumor homogenate (15).

The lower limits of quantitation for the assays were 30.0, 0.510, and 0.500 ng/mL for total antibody (TAb), conjugated dazostinag, and deconjugated dazostinag in plasma samples and 240, 4.08, and 4.00 ng/mL for TAb, conjugated dazostinag, and deconjugated dazostinag in tumor samples, respectively.

Bioanalytical results were stored in Watson LIMS (version 7.6, Thermo Fisher Scientific) and were reported to three significant figures. TAb, conjugated dazostinag, and deconjugated dazostinag concentration results below the quantitation limit (BQL) for the respective analytes were reported in Watson as BQL and were treated as zero for statistical calculations.

The plasma tumor concentrations of TAb, conjugated dazostinag, and deconjugated dazostinag reported by Frontage Laboratories, Inc. (15), were used for the PK analyses and are reported in the text, figures, and tables. All dosing is shown in payload concentration unless mentioned otherwise. PK parameters were estimated using the Phoenix WinNonlin software (version 7.0, Certara USA) with a noncompartmental approach consistent with the intravenous bolus route of administration for TAb and conjugated dazostinag and extravascular route for deconjugated dazostinag. The conjugated dazostinag equivalent dose was calculated based on Equation (A).

Conjugated‐payload equivalent dose=(ADC Dose/ADC MW)*DAR*payload MW (A)

where mTAK-500 MW = 156,000 g/mol; TAK-676 MW = 710.52 g/mol; and DAR = 3.0.

The area under the concentration–time curve (AUC) was calculated using the linear-up log-down method. The AUC was not reported for PK profiles with less than three quantifiable concentrations at consecutive time after dose. Concentrations of analytes that were BQL were set to 0.00 ng/mL for the purpose of calculating mean concentrations and the PK parameters. No statistical analyses were performed on the plasma and tumor concentration data or on derived PK parameters. All derived parameters and associated SDs are reported to three significant figures, with the exception of time, to reach maximum plasma concentration, which is reported according to the PK sampling time with no decimals. Parameters relying on the determination of the terminal elimination phase were not reported if the coefficient of determination was <0.800 or if the percentage of the AUC extrapolated to infinity observed from the time of the last observation to infinity represented >20% of the total area. AUC_0–24 hours_ was used for exposure comparison between different dosing groups.

### *In vivo* assessment of mTAK-500 efficacy in tumor-bearing mouse models

#### MC38 model

Seven-week-old female C57BL/6 mice were inoculated subcutaneously with 1.0 × 10^6^ MC38 tumor cells in the right flank. Tumor growth and body weights were monitored two times per week using Vernier calipers and the mean tumor volume (MTV) was calculated using the formula [0.5 × (length × width^2^)]. When the MTV reached approximately 80 mm^3^, animals were randomized into treatment groups (*n* = 8/group) and dosed intravenously with either vehicle (PBS), mTAK-500 at 5, 10, or 25 μg/kg as a single dose (days 0), or the isotype control antibody Kunitz trypsin inhibitor (mKTI) TAK-676, at 5, 10 or 25 μg/kg as a single dose (days 0).

Growth rate inhibition (GRI) was calculated on day 13, and tumor volume was monitored through day 23 of the study to determine the number of complete responses, defined as a decrease in tumor volume to an undetectable size (<25 mm^3^). The mean maximum body weight loss was determined for each group using the mean body weight data from the treatment period, and the mean maximum percent body weight change was calculated on the basis of pre-dose body weights.

#### CT26 model

Seven-week-old female BALB/c mice (The Jackson Laboratory, Cat. #000651) were inoculated subcutaneously with 0.8 × 10^6^ CT26 tumor cells in the right flank. Tumor growth and body weights were monitored two times per week using Vernier calipers and the MTV was calculated using the formula [0.5 × (length × width^2^)]. When the MTV reached approximately 80 mm^3^, animals were randomized into treatment groups (*n* = 8/group) and dosed intravenously with either vehicle (PBS), mTAK-500 at 5, 10 or 25 μg/kg as a single dose on study day 0.

#### Immune cell depletion experiments

Approximately seven-week-old female C57BL/6 mice were inoculated subcutaneously with 1 × 10^6^ MC38 murine colon adenocarcinoma cells in the right flank. Tumor growth was monitored twice weekly using calipers and the MTV was calculated using the formula [0.5 × (length × width^2^)]. When the MTV reached approximately 70 mm^3^ animals were randomized into treatment groups (*n* = 8/group) and treated with depletion antibodies for MDSCs (GR-1, Bio X Cell, Cat. #BE0075), macrophages (CSF1R, Bio X Cell, Cat BE0213), CD8 T cells (anti-CD8a, Bio X Cell, Cat. #BP0061), or CD4 T cells (anti-CD4, Bio X Cell, Cat. #BE0003-1) on study day −3. Relevant groups were then treated with intravenous doses of vehicle (PBS) or mTAK-500 (5 μg/kg) as a single dose on day 0.

#### Anti–PD-1 combinations

Approximately seven-week-old female C57BL/6 mice were inoculated SC with 1 × 10^6^ MC38 murine colon adenocarcinoma cells in the right flank. Tumor growth was monitored twice weekly using calipers and the MTV was calculated using the formula [0.5 × (length × width^2^)]. When the MTV reached approximately 70 mm^3^ animals were randomized into treatment groups (*n* = 8/group) and treated with intravenous doses of vehicle (PBS) or mTAK-500 (5 μg/kg) as a single dose (day 0). For the combination treatment groups, mTAK-500 dosed intravenously at 5 μg/kg was combined with anti–mPD-1 dosed IP at 10 mg/kg on a twice weekly × 2 schedule (days 0, 4). For complete responders, animals were rechallenged with 1 × 10^6^ MC38 cells in the left flank and monitored for tumor growth on both sides twice weekly. Age-matched naïve animals were inoculated in the left flank at the same time as the control.

#### Radiation combination studies

Eight-week-old female BALB/c mice were inoculated subcutaneously with 0.2 × 10^6^ CT26 tumor cells, and 8 week old female or C57BL/6 mice were inoculated subcutaneously with 1.0 × 10^6^ MC38 tumor cells. Animals were monitored for tumor growth. Once the tumors reached an average of 100 to 125 mm^3^, animals were irradiated with either mock irradiation or 8 Gy of focal beam irradiation (study day −1). Animals were then dosed intravenously with either vehicle or 5 μg/kg of mTAK-500 beginning on study day 0. Animals were monitored for tumor volumes and body weight changes twice weekly throughout the study.

### *In vivo* assessment of cytokine induction in tumor-bearing mouse models

Ten-week-old female BALB/c mice were inoculated subcutaneously with 1.0 × 10^6^ MC38 tumor cells in the right flank. When tumors grew to an estimated 300 to 500 mm^3^, animals were assigned into groups (*n* = 5/timepoint). Each group of animals received a single dose of vehicle or mTAK-500 (at 0.02, 0.06, 0.18, 0.5, 1.25, 2.5, 5.0, and 10.0 μg/kg by dazostinag payload) by intravenous injection. Plasma and tumor were collected at 6 and 24 hours after treatment.

For plasma collection, approximately 500 µL of whole blood was obtained via cardiac puncture, placed into tubes coated with K_2_EDTA (Eppendorf, Cat. #022379224) to prevent clotting and centrifuged at 10,000 rpm for 5 minutes in a Heraeus Biofuge Fresco set at 4°C. Approximately 200 µL of plasma was then transferred into U-bottom tubes and capped with a pierceable thermo plastic elastomer cap, snap-frozen on dry ice, and stored frozen at approximately −80°C. Tumor samples were excised from the mouse and placed into Covaris bags (Covaris, Cat. #520036), snap-frozen on dry ice and stored frozen at approximately −80°C.

On the day of cytokine analysis, tumor samples were dry pulverized using the cryoPREP Pulverizer according to the manufacturer’s instructions and sonicated and homogenized in 0.5% CHAPS (3-[(3-cholamidopropyl)dimethylammonio]-1-propanesulfonate, Thermo Fisher Scientific, Cat. #28299) supplemented with protease and phosphatase inhibitors (Thermo Fisher Scientific, Cat. #A32961) using the Focused-ultrasonicator. The homogenized tumor samples were then transferred to appropriately labeled 1.5-mL micro centrifuged tubes and centrifuged at 10,000 rpm in an Eppendorf Centrifuge 5417R, set at 4°C for 10 minutes. The supernatant was then transferred to a new set of labeled 1.5-mL size microcentrifuge tubes and stored on ice until quantification of cytokines, as described below. On the day of cytokine analysis, the plasma was thawed on ice prior to cytokine quantification.

Plasma and tumor samples were evaluated for the expression of IFN-α2, IP-10, TNF-α, IL-6. MCP-1, and IL-1β using the MSD U-PLEX cytokine and chemokine kits (Meso Scale Discovery Item #K15322K) according to the manufacturer’s protocol.

Standard curves for individual cytokines were generated using a five-parameter logistic equation and the concentration of individual cytokines in each sample was calculated using MSD Discovery Workbench, version 4.0.13 Desktop Edition (Meso Scale Diagnostics). The concentration below the lower limits of quantitation or above the upper limits of quantitation was assumed to be 0 for the parameter estimation.

### *In vivo* assessment of immune cell modulation in tumor-bearing mouse models

Ten-week-old female C57BL/6 mice were inoculated subcutaneously with 1.0 × 10 MC38 tumor cells (cell suspension in DMEM) in the right flank. When tumors grew to approximately 300 to 500 mm^3^, animals were randomly assigned into groups (*n* = 5/timepoint). Each group of animals received an intravenous injection of either vehicle or mTAK-500. Animals were sacrificed 3, 7, and 13 days after injection.

Tumor-draining lymph nodes (axillary, brachial, and inguinal on same side as the tumor), whole blood, and tumor tissue were collected from each study. The whole blood was collected via cardiac puncture (terminal collection) into EDTA-coated tubes and set on ice. Tumors were removed from animals and approximately 0.5 g tissue was stored in RPMI medium supplemented with 10% HI-FBS on ice until ready for processing. Lymph nodes (axillary, brachial, and inguinal on same side as tumor) were removed from animals and placed into RPMI-1640 media plus 10% HI-FBS and stored on ice until processed.

Tumor tissue was transferred to Miltenyi gentleMACS C dissociation tubes (Miltenyi Biotec, Cat. #130-036-334) supplemented with tumor dissociation enzyme mix (Miltenyi Biotec, Cat. #130-096-730) and then placed on OctoMACS tissue dissociator (Miltenyi Biotec). Samples were dissociated using the 37°C_m_TDK_1 program. Tumor cell suspensions were passed through 70-μm cell strainers (Thermo Fisher Scientific, Cat. #07-201-431). After straining, 10 mL of RPMI-1640 media supplemented with 10% HI-FBS was added to each conical tube, and cells were pelleted for 5 minutes at 300 RCF in an Allegra X-14R centrifuge set at 4°C. After removing the supernatant, the pellet was re-suspended with 1 mL of RPMI-1640 media supplemented in 10% HI-FBS and subjected for cell count. After cell count, the cells were pelleted for 10 minutes at 300 RCF in an Allegra X-14R centrifuge set at 4°C. Samples were re-suspended in RPMI-1640 media supplemented with 10% HI-FBS to obtain a final concentration of 5 × 10^7^ viable cells per mL. One hundred microliters of the single-cell suspension containing a total of 5 × 10^6^ viable cells were transferred to two 96-well U-bottom plates for immediate staining with the T-cell and macrophage/DC flow cytometry staining panels (See Supplementary Tables S9 and S10).

All lymph nodes from the same animal were then transferred into a 70-μm cell strainer placed on top of a 50-mL conical tube. A 3-mL syringe top (Thermo Fisher Scientific, Cat. #14-823-435) was used to macerate the lymph nodes completely. An additional 10 mL of RPMI-1640 media supplemented with 10% HI-FBS was added to wash each of the strainers and to ensure all cells were filtered through. The cells were then pelleted for 5 minutes at 400 RCF in an Allegra X-14R centrifuge set at 4°C. After removing the supernatant, the pellet was re-suspended with 1 mL RPMI-1640 media supplemented with 10% HI-FBS and subjected for cell count. After cell count, the cells were pelleted for 5 minutes at 400 RCF in an Allegra X-14R centrifuge set at 4°C. Samples were re-suspended in RPMI-1640 media supplemented with 10% HI-FBS to obtain a final concentration of 1 × 10^7^ viable cells per mL. One hundred microliters of the single-cell suspension containing a total of 1 × 10^6^ viable cells were transferred to two 96-well U-bottom plates for immediate staining with the T-cell and macrophage/DC flow cytometry staining panels (See Supplementary Tables S9 and S10).

Whole blood was resuspended in 10 mL of ACK lysis buffer (Thermo Fisher Scientific, Cat. #A1049201), in a 15-mL conical tube and incubated in the dark at RT for 10 to 15 minutes, shaking occasionally. Samples were then centrifuged for 5 minutes at 300 RCF in an Allegra X-14R centrifuge set at 4°C. Supernatant was removed, and the pellet was resuspended in 10 mL of RPMI-1640 media supplemented with 10% HI-FBS. The cells were then pelleted by centrifugation for 5 minutes at 300 RCF in an Allegra X-14R centrifuge set at 4°C. The supernatant was then be removed, and the pellet was re-suspended with 1 mL of RPMI-1640 media supplemented in 10% HI-FBS and cells were counted.

After counting, the cells were pelleted for 5 minutes at 300 RCF in an Allegra X-14R centrifuge set at 4°C. Samples were re-suspended in RPMI-1640 media supplemented with 10% HI-FBS to obtain a final concentration of 1 × 10^7^ viable cells per mL. One hundred microliters of the single-cell suspension containing a total of 1 × 10^6^ viable cells were transferred to two 96-well U-bottom plates for immediate flow cytometry staining with the T-cell and macrophage/DC panels (See Supplementary Tables S9 and S10).

The 96-well U-bottom plates with single-cell suspension of all samples were centrifuged for 4 minutes at 400 RCF in an Allegra X-14R centrifuge set at 4°C. The supernatant was removed and washed one time with 1× PBS.

The cells were resuspended in 100 μL of Live/Dead Fixable Dead Cell Stain at a 1:1,000 dilution in 1× PBS. The cells were incubated at RT for 10 minutes and protected from light. After incubation, cells were washed one time by adding 200 μL of 1× PBS to each well and centrifuged for 4 minutes at 400 RCF in an Allegra X-14R centrifuge set at 4°C. After centrifuging, the supernatant was discarded and the wash with 200 μL of 1× PBS was repeated twice more. The cells were then resuspended in 50 μL of Fc blocking solution containing 20 μg/mL Fc Block in 1× PBS plus 2% HI-FBS (otherwise known as “staining buffer”). The plates were incubated at approximately 4°C for 10 minutes and protected from light. After this incubation, 50 μL of a 2× surface stain mixture (comprised of the listed antibodies in Supplementary Table S9 and S10 targeting surface antigens diluted in staining buffer at twice the indicated concentrations) was added to each well. For antibodies noted as FMO controls, additional wells of tumor cells were prepared and stained with the staining mixture minus that antibody in order to aid in the gating and analysis of those markers. The cells were incubated at 4°C for 30 minutes while protected from light.

Cells were then pelleted for 4 minutes at 400 RCF in an Allegra X-14R centrifuge set at 4°C and washed twice with stain buffer. Cells were next resuspended in 200 μL of 1× Fixation/Permeabilization Working Solution from the eBioscience Foxp3/Transcription Factor Staining Buffer Set (Invitrogen, Cat. #00-5523-00) and incubated at RT for 30 minutes while being protected from light. Cells were then pelleted for 5 minutes at 400 RCF in an Allegra X-14R centrifuge set at 4°C and washed with 200 μL of 1× Permeabilization Buffer from the eBioscience Foxp3/Transcription Factor Staining Buffer Set. The wash was repeated twice.

After the permeabilization washes, 100 μL of an intracellular stain mixture was added to each well. The cells were then incubated at RT for 60 minutes while protected from light. After staining, cells were pelleted for 5 minutes at 300 RCF in an Allegra X-14R centrifuge set at 4°C and washed twice with 1× Permeabilization Buffer followed by one wash with staining buffer. Cells were then stored in 200 μL of staining buffer until ready for data acquisition. Data acquisition for each sample was performed no later than 4 days after staining is complete and was on an LSRFortessa.

Flow cytometric data files were analyzed using FlowJo software, version 10.1 (FlowJo, LLC). For the T-cell activation panel, data were gated on CD45^+^, Live/Dead negative (−) to look at changes within the immune cell compartment. These cells are presented as; % Live CD45^+^ Cells. From this gate, the total T cells (defined herein as Live/Dead dye negative/CD45^+^/CD45R^−^/CD3^+^) were further gated on CD8^+^ T cells and are reported as % CD8^+^ T cells. The CD8^+^ T cells were then separated into Ki67^+^ and Ki67^−^, and the positive gate is reported as % Ki67^+^ CD8^+^ T cells. In addition, the CD8^+^ T cells were also separated into CD69^+^ and CD69^−^, and the positive gate reported herein as % CD69^+^ CD8^+^ T cells.

Using the macrophage/DC panel, CD11c/MHCII double-positive cells were gated off live DUMP negative CD45^+^ nonneutrophils and are reported as; %CD11c^+^MHCII^+^ cells. CD11c^+^ MHCII^+^ cells were further divided into CD80^+^ and CD80^−^ populations and that data are reported as %CD80^+^ CD11c^+^ MHCII^+^ cells. See Supplementary Figs. S9 and S10 for gating strategies.

### Assessment of TAK-500 binding in human and murine cells

To assess binding and species specificity, THP1-Dual KI-R232 cells were engineered to express human CCR2 or murine CCR2 using viral transduction. Viral supernatant was collected from 293T cells 36 to 48 hours after transfection with FuGENE HD Transfection reagent (Promega, Cat. #E5911), DNA encoding human CCR2 or murine CCR2, along with Sigma-Aldrich MISSION Lentiviral Packaging Mix (MilliporeSigma, Cat. #SHP001). The viral supernatant was incubated with the target cells (THP1-Dual KI-R232 cells) overnight and placed in puromycin containing media to select cells that express human or murine CCR2.

On the day of the experiment, the cells were plated to clear, 96-well U-bottom ultralow attachment plates at 120,000 cells/100 μL per well density in FACS Staining Buffer. The cell plates were centrifuged at 400*g* for 5 minutes to pellet cells, and the supernatant was discarded by firmly flicking the plate one time onto a container. Cells were then resuspended in 50 μL of Live/Dead Fixable Near-IR Dead Cell Stain diluted 1:10,000 after resuspending it according to the manufacturer's instructions. Cells were washed by adding 200 μL of FACS Staining Buffer and centrifuging at 400*g* for 5 minutes. Cells were then resuspended in 50 μL ice-cold FACS buffer containing 10 μg/mL of Fc block and incubated for 15 minutes on ice. After Fc block, 50 μL of test article or negative controls were added to wells in a 9-point titration range with a threefold serial dilution from 15 to 0.0023 μg/mL final concentration with an additional no primary IgG control. Plates were then incubated on ice in the dark for 30 minutes. At the end of the incubation, 150 μL of FACS Staining Buffer was added to cells; plates were centrifuged at 400*g* for 5 minutes at 4°C; and the supernatant was discarded by flicking the plate one time on a container. A second wash was performed by resuspending the cells in 150 μL of FACS Staining Buffer and centrifuging at 400*g* for 5 minutes at 4°C. Cells were then resuspended in 100 μL of PE-labeled secondary antibody diluted 1:500 from supplied concentration. Cells were washed one time by adding 150 μL of FACS Staining Buffer and centrifuging at 400*g* for 5 minutes at 4°C. Cells were resuspended in 100 μL of 2% paraformaldehyde (Electron Microscopy Sciences, Cat. #1596001) in Dulbecco's phosphate-buffered saline (DPBS). Samples were then centrifuged at 400*g* for 5 minutes at 4°C and resuspended in FACS Staining Buffer. Plates were stored on ice until measured using FACS in BD FACSCanto II.

### Assessment of CCR2 expression in human tumor samples

An mQIF panel for CCR2, CD11b, and CD68, along with pan-cytokeratin (CK) to mark tumor epithelial cells and DAPI for nuclei, was established and performed as previously described (20). Tumor tissues were collected from a total of 1,108 patients with non–small cell lung cancer (NSCLC), colorectal cancer (CRC), and pancreatic ductal adenocarcinoma (PDAC) after written consent and in accordance with the principles of the Declaration of Helsinki. Inclusion criteria for samples included biopsies from patients with lung cancer, CRC, or PDAC at any stage, and exclusion criteria included a lack of tumor content in biopsy and obvious artifacts at pathology evaluation. Samples were collected between 1988 and 2012 (NSCLC), 2009 and 2017 (CRC), and 2010 and 2017 (PDAC) and assembled into tissue microarrays (TMA). TMA sections were deparaffinized, rehydrated, and incubated in antigen retrieval buffer (1 mmol/L EDTA, pH 8.0; Sigma-Aldrich) in a pressure-boiling module (LabVision) at 97°C for 20 minutes. Slides were incubated in 0.75% H_2_O_2_ in methanol at RT for 30 minutes to block endogenous peroxidase activity and subsequently in 0.3% BSA/0.05% Tween 20 blocking solution at RT for 30 minutes. Markers were detected with the following primary antibodies: mouse anti-CCR2 monoclonal IgG2a (7A7 clone, Abcam, 1:2,000), rabbit anti-CD11b monoclonal IgG (D6X1N clone, Cell Signaling Technology, 1:100), mouse anti-CD68 monoclonal IgG3k (PG-M1 clone, Dako, 1:200), all co-incubated at 4°C overnight. On day 2, secondary antibodies were added sequentially at RT for 1 hour: goat antimouse horseradish peroxidase (HRP)–conjugated IgG2a (Abcam, ab97245, 1:200), goat anti-rabbit HRP-conjugated IgG (Abcam, ab6721, 1:200), and goat antimouse HRP-conjugated IgG3 (Abcam, ab97260, 1:200). Tyramide signal amplification reagents were added after each secondary antibody corresponding to the following fluorophores in the following order: Cy3-tyramide Plus, Cy5-tyramide, and biotinylated tyramide (Akoya Biosciences) followed by Alexa Fluor 750–conjugated streptavidin (Thermo Fisher Scientific). Unbound HRPs were quenched in between secondary antibody–fluorophore pairs by treating the slides twice with 1× PBS containing 0.136 g of benzhydrazide (Sigma-Aldrich) and 50 μL 30% (w/w) hydrogen peroxide for 7 minutes at RT. Tumor epithelial cells were stained with mouse anti-CK monoclonal IgG1 conjugated with Alexa Fluor 488 (AE1/AE3 clone, eBioscience/Thermo Fisher Scientific, 1:100) at RT for 1 hour. Nuclei were counterstained with DAPI. Washes were performed on a shaking platform for 1 minute each with 1× TBS and 1× TBS-T (TBS with 0.1% Tween 20) after every application of reagent. Coverslips were mounted on slides using ProLong Gold (Invitrogen).

Stained slides were scanned under the Vectra Polaris microscope (Akoya Biosciences). Measurement of protein levels was performed using the AQUA method of mQIF (Navigate BioPharma), which allows quantification of fluorescence signal selectively within user-defined compartments based on DAPI positivity (total), CK positivity (tumor), CK negativity (stroma), or in CD11b^+^ cells (myeloid). Quantitative immunofluorescence scores were calculated dividing fluorescence intensity by the total area in a given compartment and were normalized by exposure time and bit depth as previously reported (20).

### Statistical analysis

For *in vivo* studies, the differences in the tumor growth trends over time between pairs of treatment groups were assessed by fitting each animal’s data to a simple exponential growth model and comparing the mean growth rates of the two groups. The difference in the growth rates was summarized by the GRI, which is the reduction in growth rate experienced by the treatment group relative to that of the reference group, expressed as a fraction of the vehicle growth rate. A positive GRI indicates that the tumors in the treatment group grew at a reduced rate relative to the reference group. A statistically significant *P* value (< 0.05) suggests that the trends over time for the two treatment groups were different. For all other relevant studies, the mean, SEM, and *P* value (wherein significant; relative to control) were calculated using GraphPad Prism 7 software (GraphPad Software).

### Data availability

Data are available upon reasonable request made to the corresponding authors.

## Results

### TAK-500 shows enhanced potency and improved PK compared with a nontargeted STING agonist

**Figure 1.**
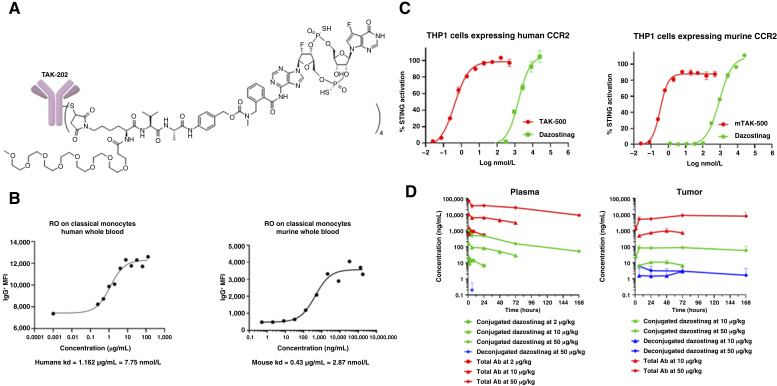
Conjugation enhances potency compared with dazostinag through selective delivery to immune cells and improved PK. **A,** Structure of STING agonist TAK-500. **B,** Receptor occupancy (RO) assessment in human (left) and murine (right) whole blood; EC_50_ values of 1.757 ± 0.524 and 1.386 ± 1.151 µg/mL, respectively. Data shown represent the mean of five human donors and five murine donors from a single experiment. Experiment was performed at least twice with consistent results between independent replicates. Error is calculated as SD. **C,***In vitro* STING activation by dazostinag in THP1 cells expressing human (left) and murine (right) CCR2. Data shown represent three technical replicates for human and two technical replicates for murine samples from a single experiment. Experiment was performed at least twice with consistent results between independent replicates. Error is calculated as SD. **D,** PK assessment of mTAK-500 in the plasma (left) and tumors (right) of MC38 tumor–bearing mice. Data shown represent three mice per group per timepoint. Error is calculated as SD.

Targeting of the STING agonist dazostinag to CCR2^+^ cells was achieved through its conjugation, via a cleavable linker, to a clinically tested anti-human IgG1 CCR2 antibody (TAK-202; Fig. 1A; refs. 21, 22). In parallel, a murine cross-reactive anti-CCR2 antibody was conjugated to generate a murine surrogate, mTAK-500, to support preclinical studies. Species-specific binding was demonstrated for both molecules (Supplementary Fig. S11). To demonstrate target engagement by TAK-500 and mTAK-500, RO was evaluated. As shown in Fig. 1B and Supplementary Tables S11 and S12, dose-dependent binding of TAK-500 and mTAK-500 to CCR2 on classical monocytes was observed in human and mouse whole blood samples, with EC_50_ values of 1.757 ± 0.524 and 1.386 ± 1.151 µg/mL, respectively. STING pathway activation was enhanced by both TAK-500 and mTAK-500 (EC_50_ = 0.4170 nmol/L and EC_50_ = 0.3393 nmol/L, respectively) compared with unconjugated dazostinag at the same molar equivalents (EC_50_ = 1,646 nmol/L and EC_50_ = 892.7 nmol/L) in human THP1 AML cell reporter cells (Fig. 1C).

An assessment of the PK properties of mTAK-500 was conducted in female C57BL/6 mice bearing MC38 murine colon adenocarcinoma syngeneic tumors (Fig. 1D; Supplementary Table S13). Total antibody and conjugated dazostinag showed more than the dose-proportional increase in plasma exposure after a single intravenous administration of mTAK-500. The deconjugated dazostinag plasma concentrations across all doses were not quantifiable. Tumor exposure to TAb and conjugated dazostinag also increased in a greater-than-dose-proportional manner after a single intravenous administration of mTAK-500. The deconjugated dazostinag exhibited extended exposure in tumor tissue and showed a less-than-dose-proportional increase between 10 and 50 μg/kg mTAK-500 doses. These data show prolonged exposure of conjugated antibody and dazostinag in the tumor, demonstrating improved PK properties intratumorally over the deconjugated STING agonist dazostinag alone (14).

### CCR2 expression and iADC-mediated immune activation *in vitro*

**Figure 2.**
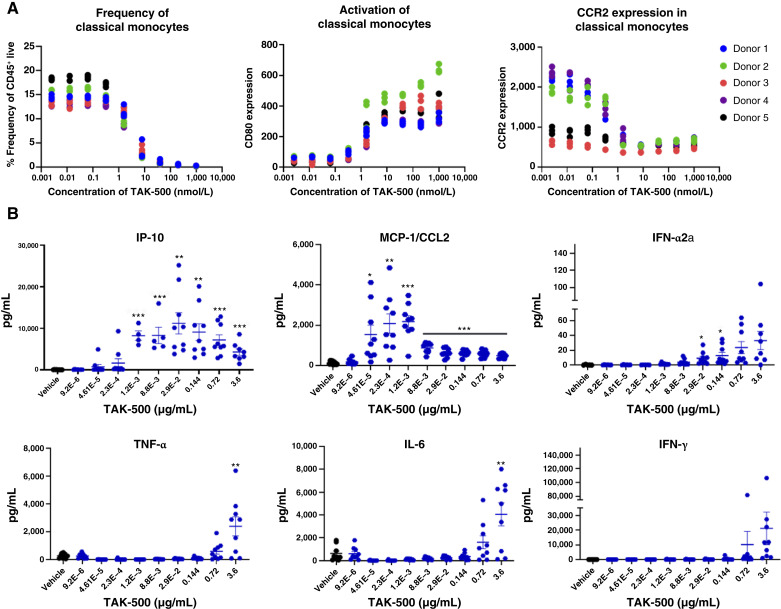
TAK-500 drives activation of monocytes and induces type-I IFN response *in vitro.***A,** Evaluation of classical monocyte frequency (left), monocyte activation (middle), and CCR2 expression (right) in PBMCs treated with TAK-500 for 24 hours. Data shown indicate the results of five human donors from a single experiment. Experiment was performed at least twice with consistent results between independent replicates. **B,** Cytokine induction in human whole blood after treatment with TAK-500 for 24 hours. Data shown represent the mean of nine human donors from a single experiment. Experiment was performed at least twice with consistent results between independent replicates. Error is calculated as SD. *P* value relative to vehicle control: *, ≤0.05; **, ≤0.01; ***, <0.00001.

Treatment with TAK-500 (at a concentration range of 0–1 µmol/L for 24 hours) resulted in dose-dependent decreases of classical monocyte frequency in human PBMCs in all donors, as well as reduction of CCR2 expression on monocytes in three of five donors (Fig. 2A). Most strikingly, TAK-500 treatment of human PBMCs resulted in a robust dose-dependent increase in the expression of the activation marker CD80 in classical monocytes across all five donors with an average EC_50_ of 1.76 nmol/L ± 0.43 (Fig. 2A). The activation of monocytes by TAK-500 was dependent on CCR2 expression (Supplementary Fig. S12A and S12B). mTAK-500 was shown to also induce CCR2-dependent activation of monocytes in a dose-dependent manner (Supplementary Fig. S12C).

We evaluated the expression of CCR2 and the impact of TAK-500 on additional immune cell populations. As shown in Supplementary Fig. S13A, although the highest levels of CCR2 protein were detected on monocytes, monocytic MDSCs (mMDSC), and macrophages, low levels of CCR2 expression were observed in CD4^+^/CD8^+^ T cells and B lymphocytes. In addition, treatment with TAK-500 resulted in the dose-dependent activation of multiple immune cell populations, including CD4^+^/CD8^+^ T cells, B cells, and NK cells, but with lower potency than in monocytes and consistent with their lower CCR2 expression (Supplementary Fig. S13B; monocyte EC_50_ = 1.76 nmol/L, CD8^+^ T-cell EC_50_ = 6.77 nmol/L, CD4^+^ T-cell EC_50_ = 9.35 nmol/L, and NK cell EC_50_ = 35.32 nmol/L).

We investigated the potential of TAK-500 to induce type-I IFNs, as well as other cytokines in human whole blood treated *in vitro*. After treatment with TAK-500 (at a concentration range of 0.0096 ng/mL to 3.6 µg/mL) for 24 hours, dose-dependent increases in IP-10, CCL2 (MCP-1), IFN-α2a, TNF-α, IL-6, and IFN-γ were observed supporting a proinflammatory effect (Fig. 2B).

As IFN-γ is expected to be a key proinflammatory driver of antitumor efficacy after immunostimulatory treatments, we characterized the immune cells that produced IFN-γ in response to TAK-500 treatment *in vitro*. As shown in Supplementary Fig. S13C, NK cells showed prominently higher levels of IFN-γ production than T cells after TAK-500 treatment of cultured PBMCs. However, isolated NK cells alone failed to produce IFN-γ when exposed to TAK-500, revealing an indirect effect of the iADC in NK-cell activation. Collectively, these data support that TAK-500 acts predominantly on myeloid cells showing high CCR2 expression and increased treatment sensitivity than other immune cell populations, but the immunostimulatory responses involve activation of additional nonmyeloid cells.

### mTAK-500 shows innate/adaptive immune activation and dose-dependent antitumor efficacy in tumor-bearing mice

**Figure 3.**
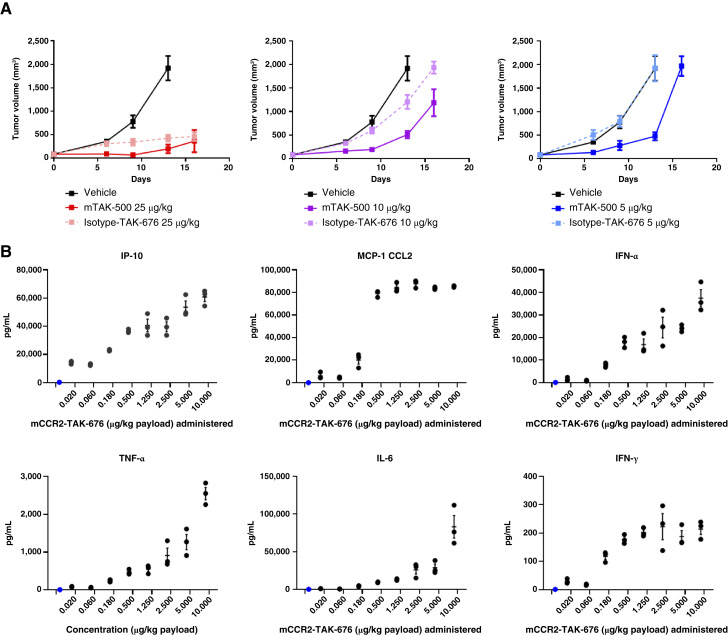
mTAK-500 shows dose-dependent efficacy and induction of cytokine release in MC38 tumor–bearing mouse models. **A,** Antitumor effect of mTAK-500 in C57BL/6 mice bearing MC38 tumors. Data shown represent the MTVs from eight mice per group. **B,** Cytokine response in the plasma of MC38 tumor–bearing mice treated with mTAK-500 at 6 hours after treatment. Blue dots represent vehicle-treated animals; black dots represent mTAK-500–treated animals. Data shown represent the mean from three mice per group. For all panels, experiment was performed at least twice with consistent results between independent replicates. For all panels, error bars indicate SEM. *P* values relative to vehicle control: *, ≤0.05; ** ≤0.01; ***, <0.00001.

To evaluate the impact of the CCR2-targeted STING iADC *in vivo*, the antitumor activity of mTAK-500 compared with both a vehicle or isotype control iADC was assessed after a single intravenous administration in C57BL/6 mice bearing established SC MC38 tumors. Treatment with mTAK-500 resulted in statistically significant tumor size reduction when dosed at 5, 10, and 25 µg/kg based on dazostinag compared with PBS control (GRI 46%, *P* = 0.001; GRI 44%, *P* < 0.001; and GRI 93%, *P* = 0.001, respectively; Fig. 3A). Higher antitumor activity of mTAK-500 was observed relative to the isotype control iADC using both 5 and 10 µg/kg (GRI −1%, *P* = 0.897 and GRI 13%, *P* = 0.093, respectively), which is striking given that the isotype control results in increased exposure relative to mTAK-500. Previous studies have shown that the continuous treatment of tumor-bearing mice using a CCR2-blocking antibody at a dose level of 45 µg/mouse resulted in only modest antitumor activity (23). Because we delivered a single iADC administration using relatively low doses (6.2, 12.4, and 31 µg/mouse of mCCR2 was administered to 5, 10, and 25 µg/kg by payload groups, respectively), we do not anticipate that the mCCR2 antibody within the iADC drives the observed antitumor effect.

We have previously demonstrated that treatment of tumor-bearing mice with unconjugated dazostinag requires doses that are at least 100-fold higher than 10 µg/kg by payload to achieve significant antitumor activity (14). This is consistent with the *in vitro* observations described above and underscores the enhancement of potency and antitumor activity achieved through the targeted delivery of STING agonism (10).

As observed in human whole blood *in vitro* (Fig. 2B), treatment of tumor-bearing mice with a single dose of mTAK-500 resulted in a dose-dependent increase in IP-10, CCL2 (MCP-1), IFN-α, TNF-α, IL-6, and IFN-γ levels *in vivo* with an average maximum increase of 205-, 813-, 304-, 377-, 4,061-, and 268-fold, respectively (Fig. 3B).

**Figure 4.**
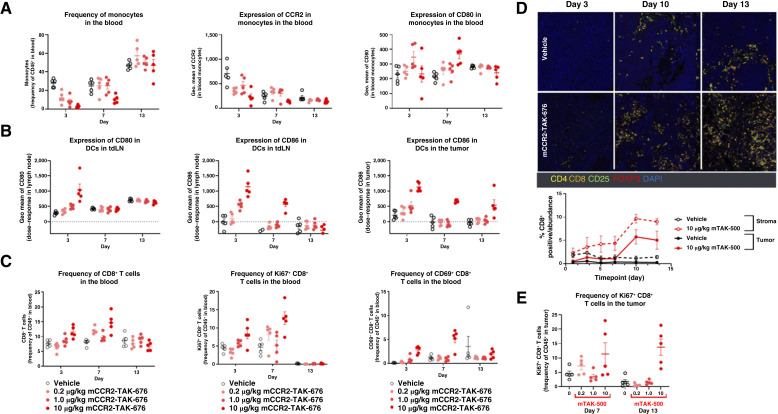
mTAK-500 shows dose-dependent induction of innate and adaptive immunity in an MC38 tumor–bearing mouse model. Data shown represent the mean from five mice per group per timepoint. Experiment was performed at least twice with consistent results between independent replicates. Error bars indicate SEM. **A,** Evaluation of monocyte frequency (left), CCR2 expression (middle), and activation (right) in the blood of MC38 tumor–bearing mice treated with mTAK-500. **B,** Evaluation of DC activation in the tumor-draining lymph nodes (tdLN; left and middle) and tumor (right) in MC38 tumor–bearing mice treated with mTAK-500. **C,** Evaluation of the frequency (left), proliferation (middle), and activation (right) of CD8^+^ T cells in the blood of MC38 tumor–bearing mice treated with mTAK-500. **D,** Evaluation of the CD8^+^ T-cell frequency (left) and proliferation (right) in the tumors of MC38 tumor–bearing mice treated with mTAK-500. **E,** Evaluation of the frequency of CD8^+^ T-cell proliferation in the tumors of MC38 tumor–bearing mice treated with mTAK-500. Geo, geometric.

We next evaluated the impact of mTAK-500 on the activation and proliferation of innate and adaptive immune cell populations in MC38 tumor–bearing mice. As observed *in vitro* with TAK-500, mTAK-500 resulted in a dose-dependent decrease in monocyte frequency (2.8% ± 0.66% in 10 µg/kg mTAK-500–treated animals vs. 27.8% ± 2.04% in vehicle-treated animals on day 3) and CCR2 expression (225.72 ± 63.13 geometric mean fluorescence intensity (gMFI) CCR2 in 10 µg/kg mTAK-500–treated animals vs. 706.40 ± 99.17 gMFI CCR2 in vehicle-treated animals) within 3 days after treatment (Fig. 4A). These effects were due to mTAK-500 mediated monocyte depletion as well as internalization of CCR2 following binding of mTAK-500 and were shown to recover to baseline levels by days 7 to 13 for all dose levels (Fig. 4A). In addition, a dose-dependent increase in monocyte activation was observed starting on day 3 and continuing through day 7 (369.00 ± 32.53 gMFI CD80 for 10 µg/kg mTAK-500–treated animals vs. 281.40 ± 4.48 gMFI CD80 for vehicle-treated animals on day 7). These results are consistent with the *in vitro* results and demonstrate the ability of the iADC to modulate CCR2-expressing monocytes in tumor-bearing mice.

DCs play a critical role in bridging innate and adaptive immune responses with potential for durable antitumor immunological memory. In DCs collected from tumor-draining lymph nodes of mTAK-500–treated animals, we observed increased expression of CD80 and CD86 3 days after treatment (1,040.20 ± 188.83 gMFI CD80 and 1,143.20 ± 146.49 gMFI CD86 in 10 µg/kg mTAK-500–treated animals vs. 278.40 ± 24.25 gMFI CD80 and 3.28 ± 97.01 gMFI CD86 in vehicle-treated animals; Fig. 4B). There was also a dose-dependent increase in CD86 expression on intratumoral DCs 3 days after treatment with greatest increase observed at 10 µg/kg mTAK-500 (1,134.80 ± 65.24 gMFI CD86 in 10 µg/kg mTAK-500–treated animals vs. 212.68 gMFI CD86 in vehicle-treated animals; Fig. 4B).

To assess whether treatment with mTAK-500 resulted in subsequent activation or amplification of adaptive immune responses, the proliferation and activation of CD8^+^ T cells in whole blood was evaluated. Increased frequency (14.76% ± 1.48% in 10 µg/kg mTAK-500–treated animals vs. 8.03% ± 0.49% in vehicle-treated animals on day 7), proliferation (as assessed by Ki67, 12.68% ± 1.74% in 10 µg/kg mTAK-500–treated animals vs. 4.65% ± 0.83% in vehicle-treated animals on day 7), and activation (as assessed by CD69, 5.19% ± 0.86% in 10 µg/kg mTAK-500–treated animals vs. 1.19% ± 0.23% in vehicle-treated animals on day 7) of CD8^+^ T cells was observed within whole blood on days 3 and 7 after treatment with mTAK-500 (Fig. 4C).

Intratumoral CD8^+^ T cells increased after 10 days of treatment with mTAK-500 and continued to increase through day 13 (Fig. 4D). An increase in Ki67^+^ CD8^+^ T cells was found on days 7 and 13 after treatment with the most pronounced effects observed after treatment with 10 µg/kg mTAK-500 (13.75% ± 2.82% in 10 µg/kg mTAK-500–treated animals vs. 1.71% ± 0.79% in vehicle-treated animals on day 13; Fig. 4E), supporting a favorable immunostimulatory effect of the iADC on intratumoral effector T cells.

The ability of mTAK-500 to drive antitumor efficacy via an innate-to-adaptive immune cell engagement was further supported by the selective elimination of MDSCs, tumor-associated macrophages (TAM), DCs, and CD4^+^ or CD8^+^ T cells prior to treatment with mTAK-500. As shown in the Supplementary Fig. S14A–S14E, the antitumor effect of mTAK-500 was significantly reduced by the absence of DCs, CD8^+^ T cells, or CD4^+^ T cells but not by the depletion of MDSCs or TAMs. Collectively, these results indicate that mTAK-500 mediates its antitumor effect via local and systemic immunostimulatory responses requiring both innate and adaptive immune cell populations.

### mTAK-500 shows enhanced efficacy and increased immune activation when combined with αPD-1 or radiation

**Figure 5.**
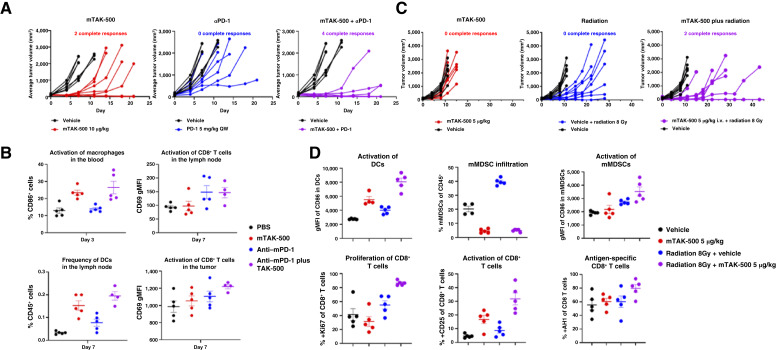
mTAK-500 shows durable enhanced efficacy and increased activation of innate and adaptive immune responses when combined with anti–PD-1 or radiation. **A,** Antitumor effect of mTAK-500 with and without αPD-1 therapy in C57BL/6 mice bearing MC38 tumors. Data shown represent the MTVs from eight mice per group. **B,** Evaluation of innate and adaptive immune-cell activation in the blood, lymph nodes, and tumors of MC38 tumor–bearing mice treated with mTAK-500 with and without αPD-1. Data shown represent the mean from five mice per group per timepoint. **C,** Antitumor effect of mTAK-500 with and without 8 Gy of focal radiation treatment in BALB/c mice bearing CT26 tumors. Data shown represent the MTVs from eight mice per group per timepoint. **D,** Evaluation of innate and adaptive immune cell frequency and activation in the tumors of MC38 tumor–bearing mice treated with mTAK-500 with and without 8 Gy of focal irradiation. Data shown represent the mean for five mice per group per timepoint. For all panels, experiment was performed at least twice with consistent results between independent replicates. In all panels, error bars indicate SEM.

The presence and expansion of functional antitumor effector T cells is a critical factor in conferring antitumor efficacy to T-cell checkpoint inhibitor therapy ICIs, such as anti–PD-1 and anti–PD-L1 antibodies (24, 25). Activation of the STING pathway and subsequent CD8^+^ T-cell infiltration resulting from mTAK-500 administration may therefore result in additive and/or synergistic effect. Accordingly, combining mTAK-500 with anti–PD-1 treatment demonstrated enhanced antitumor efficacy (GRI 110%; *P* < 0.001) compared with mTAK-500 or anti–PD-1 alone in MC38 tumor–bearing mice (GRI 76%; *P* = 0.001 and GRI 33%; *P* = 0.013, respectively; Fig. 5A).

Combining anti–PD-1 with mTAK-500 slightly increased the activation of macrophages in the blood of treated animals (26.44% ± 3.64% in anti–PD-1 plus mTAK-500–treated animals vs. 23.40% ± 1.46% in mTAK-500–treated animals; Fig. 5B). In addition, there was a higher frequency of DCs in the tumor-draining lymph nodes (0.20% ± 0.02% in anti–PD-1 plus mTAK-500–treated animals vs. 0.15% ± 0.02% in mTAK-500–treated animals; Fig. 5B) with comparable CD8^+^ T-cell activation relative to controls (Fig. 5B). By contrast, increasingly activated CD8^+^ tumor-infiltrating lymphocytes (TIL) were observed in the TME after treatment with the iADC (as assessed by CD69, 1,219.75 ± 25.46 gMFI in anti–PD-1 plus mTAK-500–treated animals vs. 1,053.80 ± 58.06 gMFI in mTAK-500–treated animals; Fig. 5B), supporting that the combination of anti–PD-1 with mTAK-500 led to increased efficacy mediated, at least in part, to enhanced T cell–mediated tumor elimination.

Radiation can prime adaptive antitumor responses by enhancing the cross-presentation of tumor-associated antigens via multiple mechanisms, including increased antigen release and upregulation of MHCI expression. Based on this, we hypothesized that mTAK-500 may show enhanced efficacy in combination with localized radiation treatment. As MC38 tumor–bearing mice show significant response to localized radiation treatment alone, combination studies with radiation were evaluated using a CT26 tumor mouse model. As shown in Fig. 5C, combining low-dose mTAK-500 (5 µg/kg) with 8-Gy radiation enhanced the efficacy in CT26 tumor–bearing mice (GRI 88%; *P* < 0.001) when compared with either mTAK-500 or radiation alone (GRI 8%; *P* = 0.2 and GRI 68%; *P* < 0.001, respectively).

Treatment with mTAK-500 plus radiation resulted in increased activation of intratumoral DCs (8,065.60 ± 514.90 gMFI CD86 in radiation plus mTAK-500–treated animals vs. 5,522.00 ± 439.74 gMFI CD86 in mTAK-500–treated animals; Fig. 5D) associated with a reduction in mMDSCs similar to that observed with mTAK-500 alone (4.95% ± 0.41% in radiation plus mTAK-500–treated animals vs. 4.60% ± 0.65% in mTAK-500–treated animals; Fig. 5D). The combination treatment also increased the activation of the remaining mMDSCs 3 days after dosing (3,524.60 ± 349.31 gMFI CD86 in radiation plus mTAK-500–treated animals vs. 2,177.00 ± 311.54 gMFI CD86 in mTAK-500–treated animals; Fig. 5D) and increased the activation and proliferation of CD8^+^ TILs 7 days after treatment initiation (as assessed by CD25 for activation: 31.78% ± 4.35% in radiation plus mTAK-500–treated animals vs. 16.79% ± 3.00% in mTAK-500–treated animals; and Ki67 for proliferation: 83.36% ± 1.51% in radiation plus mTAK-500–treated animals vs. 31.40% ± 6.96% in mTAK-500–treated animals; Fig. 5D). There was an increase in the levels of AH tetramer–positive tumor antigen–specific CD8^+^ TILs 7 days after treatment with combination therapy that was more pronounced than it was after treatment with mTAK-500 or PD-1 blockade alone (79.74% ± 5.61% in radiation plus mTAK-500 vs. 60.41 ± 4.61 in mTAK-500–treated animals and 55.36 ± 7.51 in vehicle-treated animals; Fig. 5D). These data demonstrate that combining mTAK-500 with localized radiation results in a favorable TME immune composition, ultimately driving increased efficacy in tumor-bearing mice.

### High CCR2 expression is associated with enhanced local adaptive immune responses and specific clinical/molecular features in human tumors

**Figure 6.**
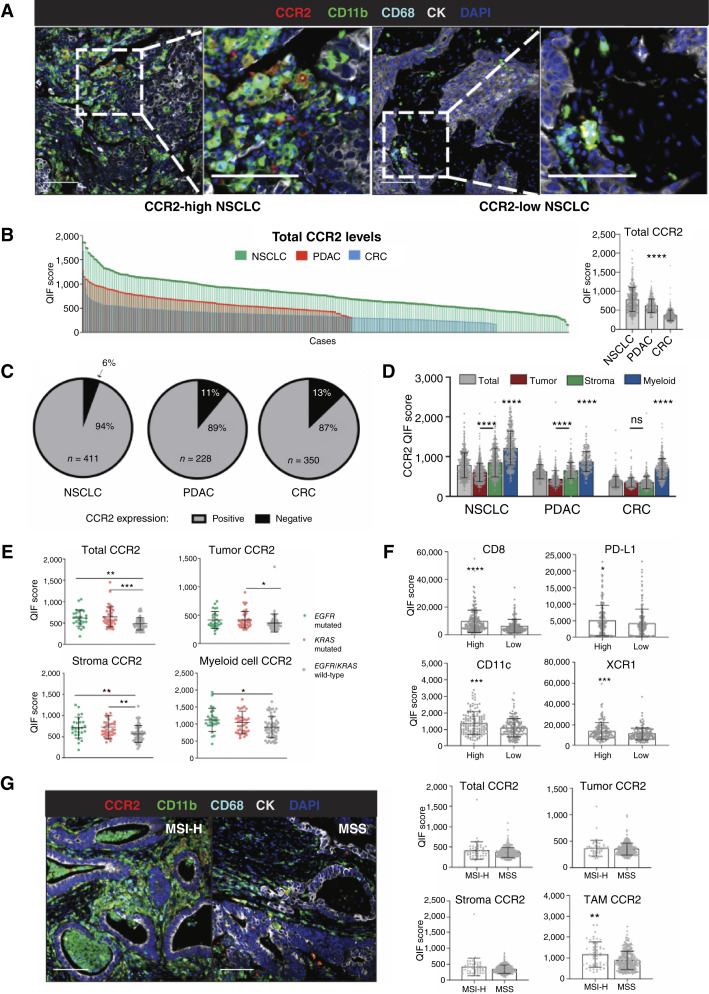
Spatially resolved and quantitative analysis of CCR2 in human NSCLC, PDAC, and CRC. **A,** Representative multicolor fluorescence micrographs of NSCLC cases with high (left) and low CCR2 protein expression (right). The color code for the markers is indicated within the caption. CCR2 colocalizes with CD11b/CD68 double-positive myeloid cells both within the CK^+^ tumor epithelium and in the surrounding stroma. Scale bar, 250 µm. **B,** Expression and distribution of total CCR2 protein levels (all cells) in NSCLC (green bars), PDAC (red bars), and CRC (blue bars). Each column in the histogram indicates the total CCR2 protein level in an individual tumor from the cohort. The chart on the right shows the mean levels of CCR2 protein across the three tumor types. **C,** Detection of CCR2 positivity in the tumor samples using the visual threshold showed a detectable signal in 94% of NSCLCs (*N* = 411), 89% of PDACs (*N* = 228), and 87% of CRCs (*N* = 350). **D,** Levels of CCR2 protein measured in the entire tumor tissue sample (total), or selectively in CK^+^ tumor epithelial cell areas (tumor), in CK^−^ tumor tissue areas (stroma) and in CD11b^+^ myeloid cells (myeloid) across NSCLC, PDAC, and CRC. **E,** Levels of CCR2 protein in selected tumor tissue compartments across primary lung adenocarcinomas with activating *EGFR* (green, *n* = 26) and *KRAS* mutations (red, *n* = 38) and in tumors lacking oncogenic variants in both oncogenes (gray, *n* = 57). Each dot in the graph represents the score in an individual tumor sample. **F,** Levels of CD8, PD-L1, CD11c, or XCR1 protein in primary NSCLCs with low or high CCR2 protein levels. Cases were stratified into high/low groups using the median total CCR2 protein scores. **G,** Representative multicolor fluorescence micrographs of CCR2, tumor, and myeloid cells in CRC cases with microsatellite instability–high (MSI-H; left) and microsatellite stable (MSS) status (right). The color code for the markers is indicated within the caption. The charts on the right show the CCR2 protein levels across the MSI-H (*n* = 51) and MSS (*n* = 282) CRC subgroups in the entire tissue area (total) or selectively measured in CK^+^ epithelial tumor cells (tumor), CK^−^ stromal cells (stromal), or in CD68^+^ TAMs. *, *P* < 0.05; **, *P* < 0.01; ***, *P* < 0.001; ****, *P* < 0.0001; ns, not significant with *P* > 0.05. QIF, quantitative immunofluorescence.

Evaluation of multiple immunocompetent syngeneic tumor-bearing mouse models demonstrated that the baseline levels of CCR2-expressing intratumoral mMDSCs were positively associated with antitumor response to mTAK-500 (*R*^2^ = 0.87; *P* = 0.006; Supplementary Fig. S15). Based on these observations, we evaluated the prevalence and localized expression pattern of CCR2 protein in human tumors using mQIF panels containing the markers DAPI, CK, CCR2, CD11b, and CD68 in >1,000 primary human tumors including NSCLC (two cohorts, *N* = 411), CRC (two cohorts, *N* = 350), and PDAC (one cohort, *N* = 228) represented in tissue microarrays (Supplementary Table S14). As shown in Fig. 6A, this analysis confirmed the predominant expression of CCR2 with a membranous-like staining pattern in stromal cells with morphology and immunophenotype consistent with intratumoral myeloid cells, lower expression in other nonmyeloid stromal cell populations, and the virtual absence of signal in malignant tumor epithelial cells. The quantitative analysis of CCR2 protein levels across tumor types showed a wide range of scores with consistently higher expression in NSCLC samples, followed by PDAC samples, and lower levels in CRC samples (Fig. 6B). In addition, using the visual detection threshold, CCR2 protein expression was identified in 94% of NSCLC samples, 89% of PDAC samples, and 87% of CRC samples (Fig. 6C). The quantitative analysis of CCR2 protein signal in marker-defined tumor tissue compartments showed significantly higher levels in CD11b^+^ myeloid cells than in CK^−^ (nontumor) stromal areas and CK^+^ cancer cell nests; and this was consistent across the different tumor types analyzed (Fig. 6D). In addition, high CCR2 protein levels were higher in primary lung adenocarcinomas harboring activating mutations in *EGFR* or *KRAS* than in EGFR/KRAS wild-type tumors, and this difference was more pronounced when measuring CCR2 selectively in the stromal and CD11b^+^ myeloid cell areas than within the cancer cell compartments that contain a limited number of infiltrating CCR2-expressing myeloid cells (Fig. 6E). In addition, the CCR2 protein levels of NSCLCs were positively associated with increased CD8^+^ TILs, local PD-L1 expression, and elevated levels of the type-1 conventional type DC markers CD11c and XCR1, supporting their association with both productive local adaptive immune responses and PD-L1–mediated immune evasion as shown in Fig. 6F. In CRC samples, higher levels of myeloid cell CCR2 were seen in tumors harboring mismatch repair deficiency/microsatellite instability–high than in microsatellite stable carcinomas, and this difference was predominant when measuring CCR2 in CD68^+^ TAMs (Fig. 6G). Collectively, these data reveal that CCR2 is highly expressed in intratumoral myeloid cells on the majority of primary NSCLC, PDACs, and CRCs and that high CCR2 is associated with enhanced local adaptive immune responses and specific clinical and molecular tumor subsets.

## Discussion

In this study, we present the first report of the generation and testing of biological activity, mechanism of action, and antitumor effect of a human CCR2-targeted STING iADC, TAK-500. Furthermore, a murine surrogate mTAK-500 was generated to characterize the impact of a CCR2-STING iADC *in vivo*. We demonstrate that the selective activation of the STING pathway in CCR2-expressing myeloid cells resulted in enhanced potency in STING agonism compared with the unconjugated STING agonist dazostinag alone. These observations are particularly interesting, as treatment with anti-CCR2 alone had little effect on tumor regression (23).

Furthermore, when administered systemically to MC38 tumor–bearing mice, mTAK-500 demonstrated dose-dependent antitumor activity with doses that are at least 100 times lower than those required to achieve comparable efficacy using dazostinag. Additionally, a single dose of mTAK-500 activated innate and adaptive immune cells in the blood, lymph nodes, and TME of MC38 tumor–bearing mice. We observed transient depletion of circulating CCR2-expressing cells, such as monocytes and MDSCs, and increased activation of DCs and effector T cells in tumor-bearing mice after treatment with mTAK-500. As depletion of key immune cell populations, such as DCs or CD8^+^ T cells, could be counterproductive to driving antitumor activity, it is important to note that this is transient and observed only in the periphery and not in the TME of tumor-bearing mice and that the depletion of MDSCs and TAMs did not impact mTAK-500–driven efficacy in tumor-bearing mice. Collectively these data highlight the orchestration of multiple immune cell types through a balance of TAK-500–induced depletion, restoration, and activation with sufficient magnitude and duration to drive antitumor activity. Together, our results reveal a prominent and favorable immunomodulatory effect of TAK-500 including both innate and adaptive immune cells associated with prominent antitumor responses and positive interactions with other anticancer treatments. This supports a strong clinical potential of this agent as an anticancer treatment and provides proof-of-principle data to support the selective modulation of immune cell populations using iADCs, a novel approach to anticancer immunotherapy with possible implications for other immunologic diseases.

We also show prominent expression of CCR2 protein in intratumor myeloid cells from the vast majority of human carcinomas from different locations including lung, colorectal, and pancreatic malignancies. The high expression of CCR2 found in the intratumor myeloid cells from NSCLC, CRC, and PDAC support a possible role of the iADC to treat patients with these highly prevalent malignancies. These results also suggest the possibility to select patients with high baseline expression of CCR2 in myeloid cells who may be more likely to benefit from the treatment. The prominent expression of CCR2 in specific clinicopathologic/molecular subgroups of lung adenocarcinomas harboring oncogenic mutations in EGFR and KRAS, as well as in CRCs with microsatellite instability–high status further support the potential for refined patient selection. Of note, patients with *EGFR* mutations and a fraction of those with activating KRAS mutant NSCLC show limited benefit from currently available ICIs, which makes these populations interesting candidates for testing the antitumor effect of the CCR2-STING iADC alone or in combination with other targeted therapies.

Our study has several limitations. Although comparable *in vitro* activity of TAK-500 and mTAK-500 was established, the *in vivo* experiments were conducted solely using a murine surrogate iADC. Therefore, although it demonstrates the potential of a CCR2-targeted STING iADC, it may not reflect the same biology elicited by TAK-500 in humans. Additionally, mouse models have limitations in recapitulating human disease and may not accurately reflect more complex human tumors evolving over longer time periods. We are also continuing to explore the best approach to understand the relative contribution of CCR2^+^ monocytes in the blood versus tumor myeloid cells in our observed responses. Although the use of tissue microarrays has the potential to under- or overrepresent the markers assessed when evaluated in small cohorts, in the present study, we evaluated hundreds of samples to increase our confidence in our observations. The comparable results seen within cohorts and across different malignancies support the consistency of our findings. Finally, although these studies identify CCR2 expression as a potential determinant of activity, it is important to note that other factors may also contribute to the activity of TAK-500 (or the murine surrogate).

In this study, we have shown that TAK-500 achieves enhanced STING agonism and proinflammatory responses via improved PK and selective delivery to CCR-expressing cells. Furthermore, we demonstrated that the iADCs TAK-500 and mTAK-500 induce CCR2-dependent innate and adaptive immune cell activation and prominent antitumor effect in both human cells and tumor-bearing mice, wherein CCR2 is highly expressed in intratumoral myeloid cells from human malignancies and high CCR2 is associated with local adaptive immune responses and specific clinical and molecular tumor subsets.

The mechanism of action of TAK-500 involves the selective depletion of CCR2^+^ myeloid cells, the favorable polarization of macrophages toward a proinflammatory phenotype, enhanced activation of DCs, and the mobilization of CD8^+^ TILs. These responses are augmented by the concurrent use of PD-1 axis blockers or radiation therapy in animal models, supporting the potential for enhanced efficacy for TAK-500 combination therapies.

In recent years, there have been prominent breakthroughs in the use of cancer cell–directed ADCs to treat patients with advanced solid tumors. Based on these findings, there has been an increase in the number of linker–payloads being evaluated over the past 5 years with new technologies and approaches being developed to address specific limitations of previously tested ADCs in the clinic (26–28). However, to the best of our knowledge, the therapeutic exploitation of selectively targeting active payloads to target immune cells has not yet been explored. The iADC TAK-500 represents an innovative approach that has the potential to enhance innate immunity and the local adaptive antitumor immune responses with a favorable therapeutic index. Moreover, the concept of iADCs could be further expanded to include targeting of other cells or multiple cell types in the TME using multivalent antibodies and/or using different payloads to provide even greater antitumor potential in the future.

## Supplementary Material

Supplementary Figure 1 Analysis of TAK-500 iADC.

Supplementary Figure 2 Analysis of mTAK-500 iADC.

Supplementary Figure 3 Gating Strategy for Receptor Occupancy in Murine Whole Blood

Supplementary Figure 4 Gating Strategy for Receptor Occupancy in Human Whole Blood

Supplementary Figure 5 Gating Strategy for Monocyte Activation in PBMCs

Supplementary Figure 6 Gating Strategy for Evaluating CCR2 in Dissociated Tumor Cells: T Cell Panel

Supplementary Figure 7 Gating Strategy for Evaluating CCR2 in Dissociated Tumor Cells: Monocyte Panel

Supplementary Figure 8 Gating Strategy for Evaluating T and NK Cell Activation

Supplementary Figure 9 Gating Strategy for Murine T Cell Panel

Supplementary Figure 10 Gating Strategy for Murine Macrophage/Dendritic Cell Panel

Supplementary Figure 11 Binding Curves Demonstrate Species Specificity for antibody portions of TAK-500 and mTAK-500

Supplementary Figure 12 Complete loss of receptor-mediated TAK-500 and mTAK-500 activity observed on human and murine peripheral blood monocytes lacking cell surface CCR2 expression

Supplementary Figure 13 Evaluation of CCR2 expression levels and the impact of TAK-500 treatment on T and NK cell activation and cytokine production in vitro.

Supplementary Figure 14 mTAK-500 induced efficacy requires CD8+ T cell mediated adaptive immunity.

Supplementary Figure 15 Evaluation of CCR2 expression in intratumoral mMDSCs within syngeneic tumor bearing mouse models via flow cytometry correlated with the antitumor response of those models to mTAK-500. Models evaluated include C1498, B16F10, MC38, H22, CT26, JC, and Panc02.

Supplementary Table 1 Cell Lines Utilized Within These Studies

Supplementary Table 2 Example Conditions for generating QTOF- liquid chromatography/mass spectrometry (LCMS) spectra

Supplementary Table 3 Antibodies Used in Murine Receptor Occupancy Flow Panel

Supplementary Table 4 Antibodies Used in Human Receptor Occupancy Flow Panel

Supplementary Table 5 Antibodies Used in Monocyte Activation Flow Panel

Supplementary Table 6 T Cell Panel for Evaluating Dissociated Tumor Cells

Supplementary Table 7 Monocyte Panel for Evaluating Dissociated Tumor Cells

Supplementary Table 8 Flow Panel for T and NK Cell Activation in Human PBMCs

Supplementary Table 9 Antibodies Used in T Cell Panel

Supplementary Table 10 Antibodies Used in Macrophage/Dendritic Cell Panel

Supplementary Table 11 TAK-500 enhances type I IFN response in THP1 cells expressing human CCR2

Supplementary Table 12 mTAK-500 enhances type I IFN response in THP1 cells expressing murine CCR2

Supplementary Table 13 Pharmacokinetic Parameters of Total Ab, Conjugated Dazostinag, and Deconjugated Dazostinag in Female C57BL/6 Mice Bearing MC38 Tumors After Intravenous Administration of mTAK-500 at 2, 10, and 50 µg/kg

Supplementary Table 14 Clinicopathologic characteristics of NSCLC, CRC and PDAC cohorts
