# Supplementary material for: Selective STING Activation in Intratumoral Myeloid Cells via CCR2-Directed Antibody–Drug Conjugate TAK-500
Source: Cancer Immunol Res. 2025 Feb 7;13(5):661–79. doi: 10.1158/2326-6066.CIR-24-0103 (PMC12046323; doi:10.1158/2326-6066.CIR-24-0103)

**Supplementary Figure 1:** Analysis of TAK-500 iADC. A. TAK-500 Hydrophobic interaction chromatography (HIC). B. TAK-500 Size exclusion chromatography (SEC). C. TAK-500 LC-QTOF Heavy Chain. D. TAK-500 LC-QTOF Light Chain


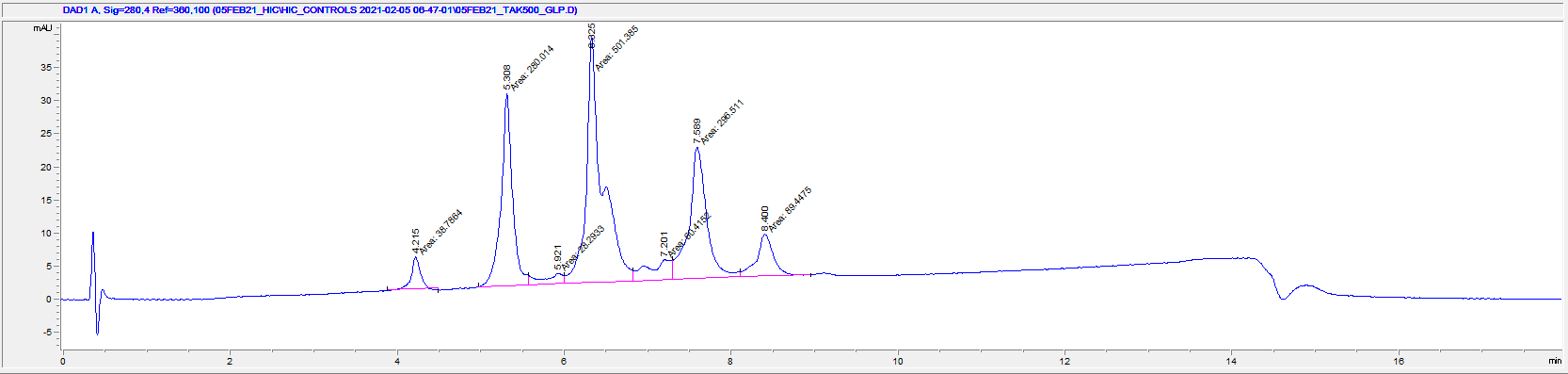
**A.**

**B.**


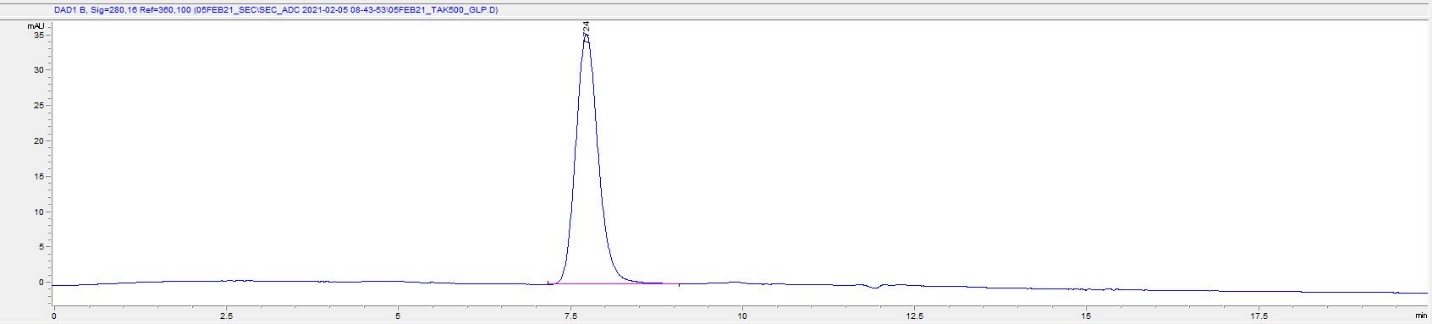


**C.**


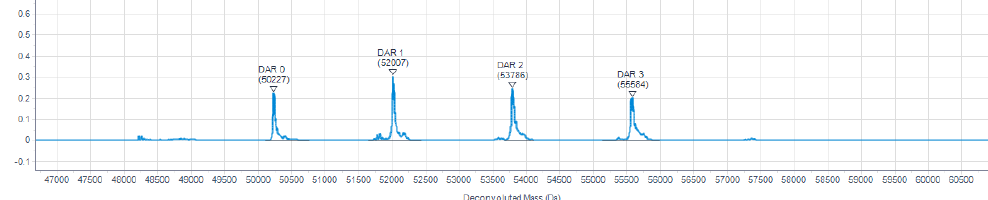


**D.**


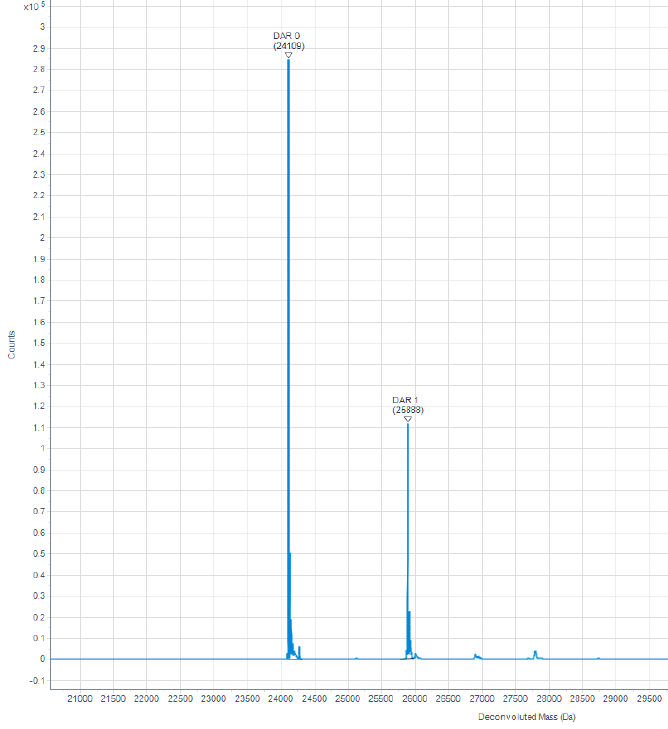

Supplement: Supplementary Figure 1 — Analysis of TAK-500 iADC. [file cir-24-0103_supplementary_figure_1_supps1.docx]
