# Supplementary material for: Selective STING Activation in Intratumoral Myeloid Cells via CCR2-Directed Antibody–Drug Conjugate TAK-500
Source: Cancer Immunol Res. 2025 Feb 7;13(5):661–79. doi: 10.1158/2326-6066.CIR-24-0103 (PMC12046323; doi:10.1158/2326-6066.CIR-24-0103)

**Supplementary Figure 2:** Analysis of mTAK-500 iADC. A. mTAK-500 Hydrophobic interaction chromatography (HIC). B. mTAK-500 Size exclusion chromatography (SEC). C. mTAK-500 LC-QTOF Heavy Chain. D. mTAK-500 LC-QTOF Light Chain.

**A.**


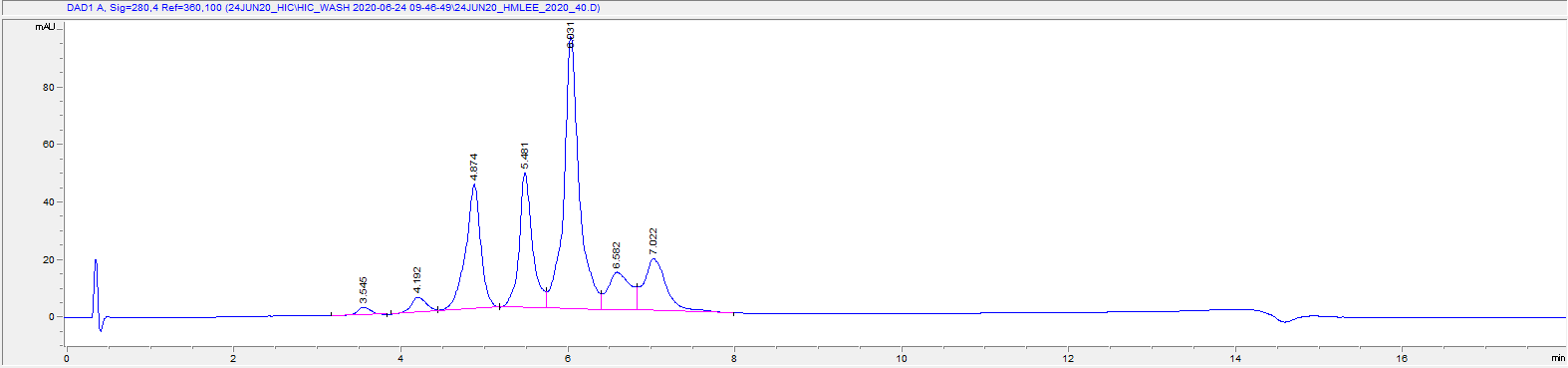


**B.**


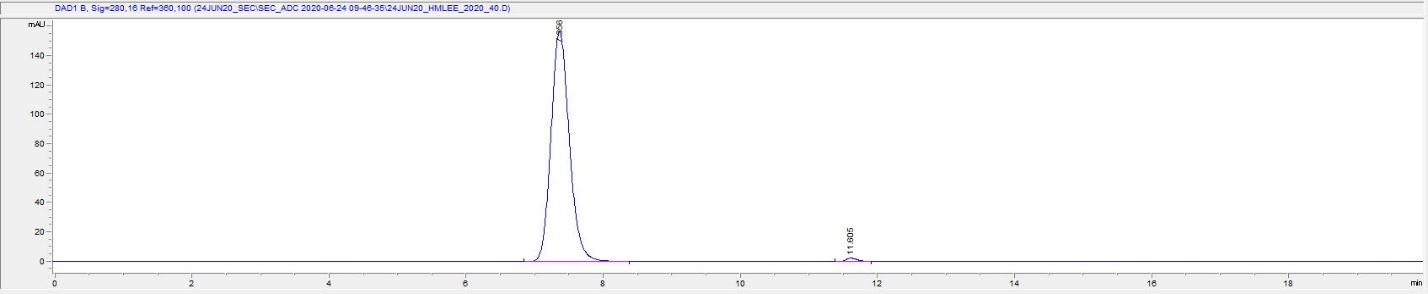


**C.**


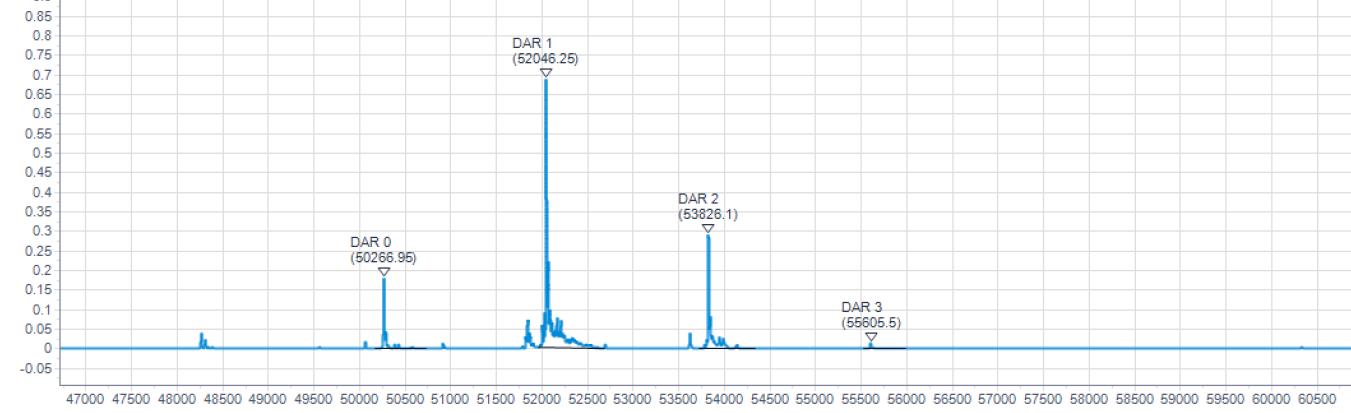


**D.**


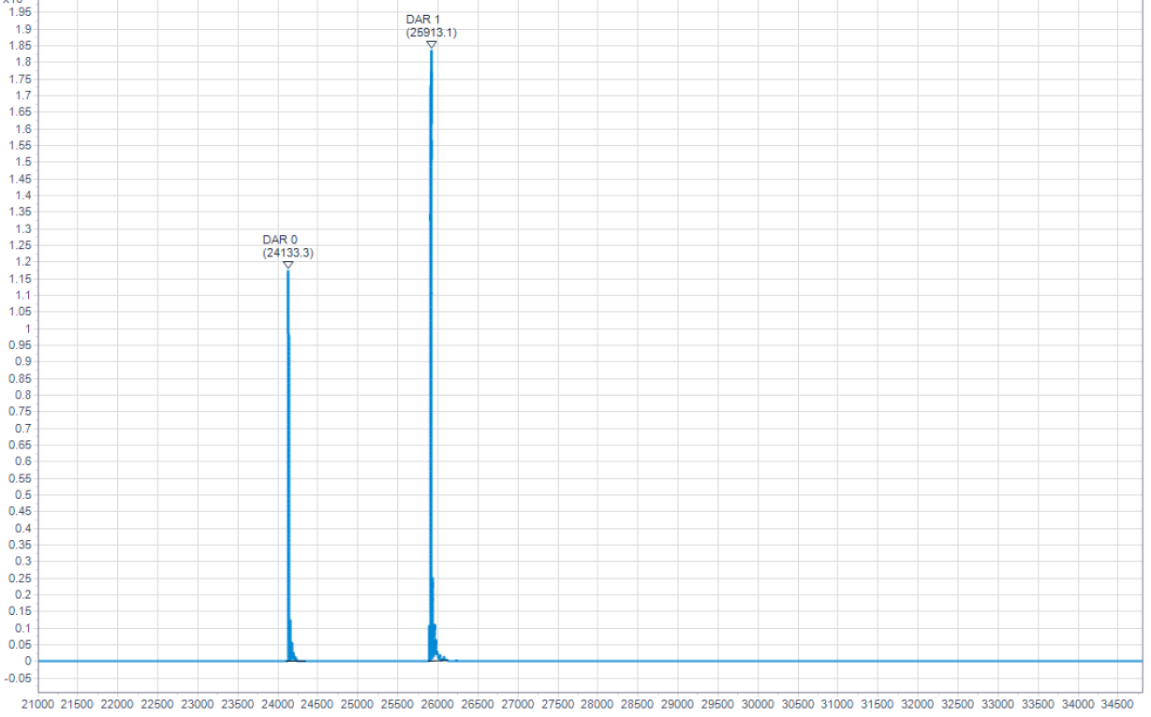

Supplement: Supplementary Figure 2 — Analysis of mTAK-500 iADC. [file cir-24-0103_supplementary_figure_2_supps2.docx]
