# Supplementary figures and images for: Selective STING Activation in Intratumoral Myeloid Cells via CCR2-Directed Antibody–Drug Conjugate TAK-500
Source: Cancer Immunol Res. 2025 Feb 7;13(5):661–79. doi: 10.1158/2326-6066.CIR-24-0103 (PMC12046323; doi:10.1158/2326-6066.CIR-24-0103)

**Supplementary Figure 3.** Gating Strategy for Receptor Occupancy in Murine Whole Blood

**
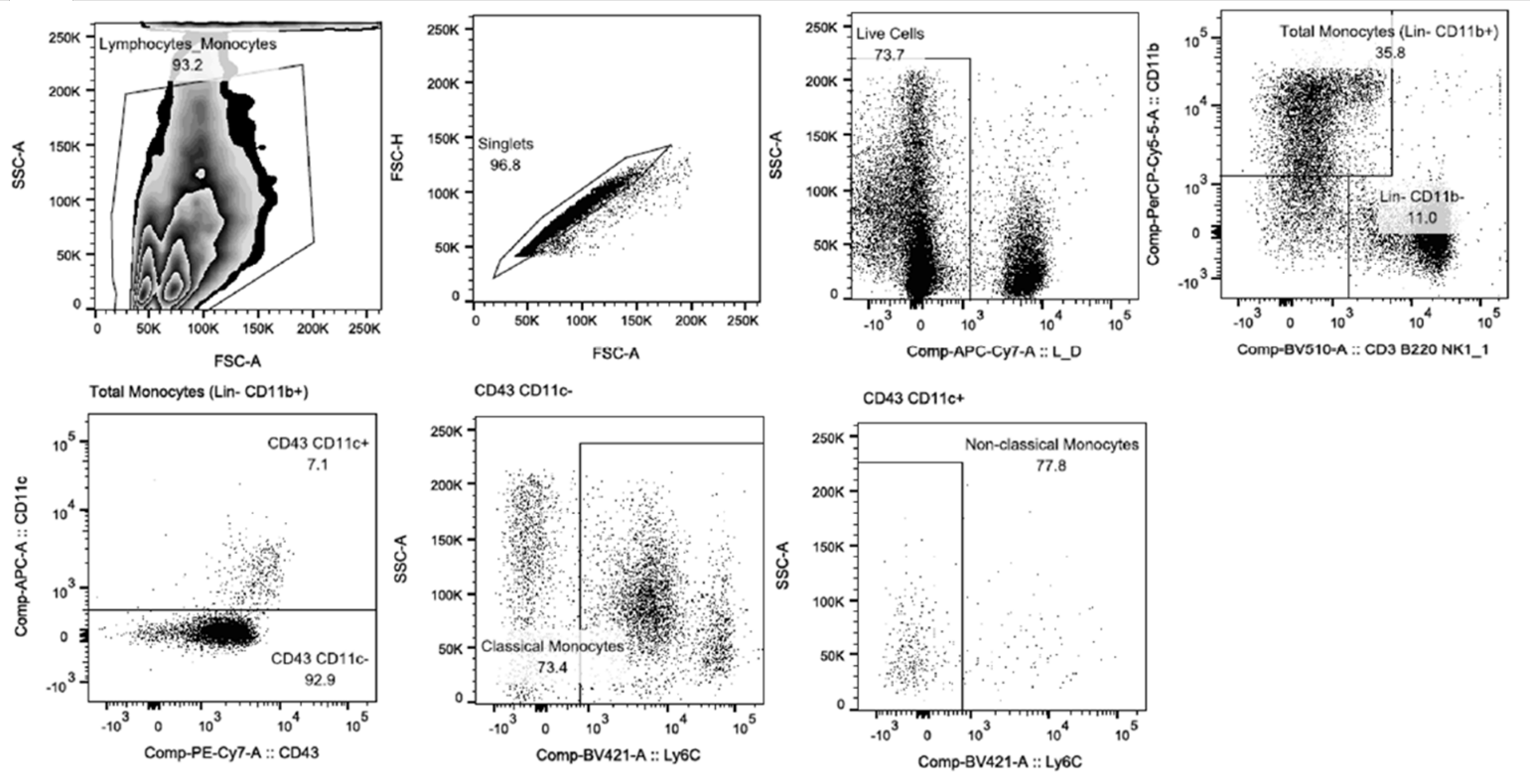
**

Supplement: Supplementary Figure 3 — Gating Strategy for Receptor Occupancy in Murine Whole Blood [file cir-24-0103_supplementary_figure_3_supps3.docx]

**Supplementary Figure 4.** Gating Strategy for Receptor Occupancy in Human Whole Blood

**
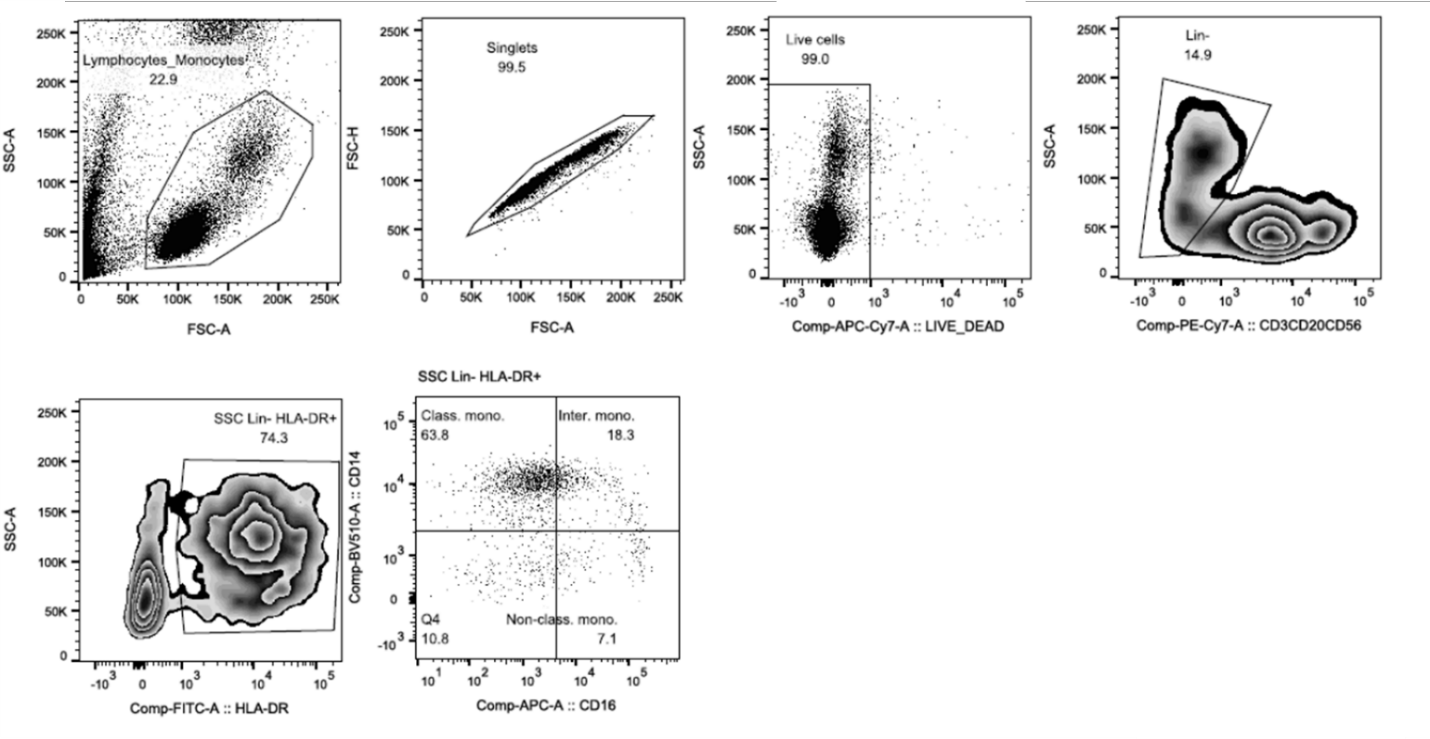
**

Supplement: Supplementary Figure 4 — Gating Strategy for Receptor Occupancy in Human Whole Blood [file cir-24-0103_supplementary_figure_4_supps4.docx]

**Supplementary Figure 5.** Gating Strategy for Monocyte Activation in PBMCs

**
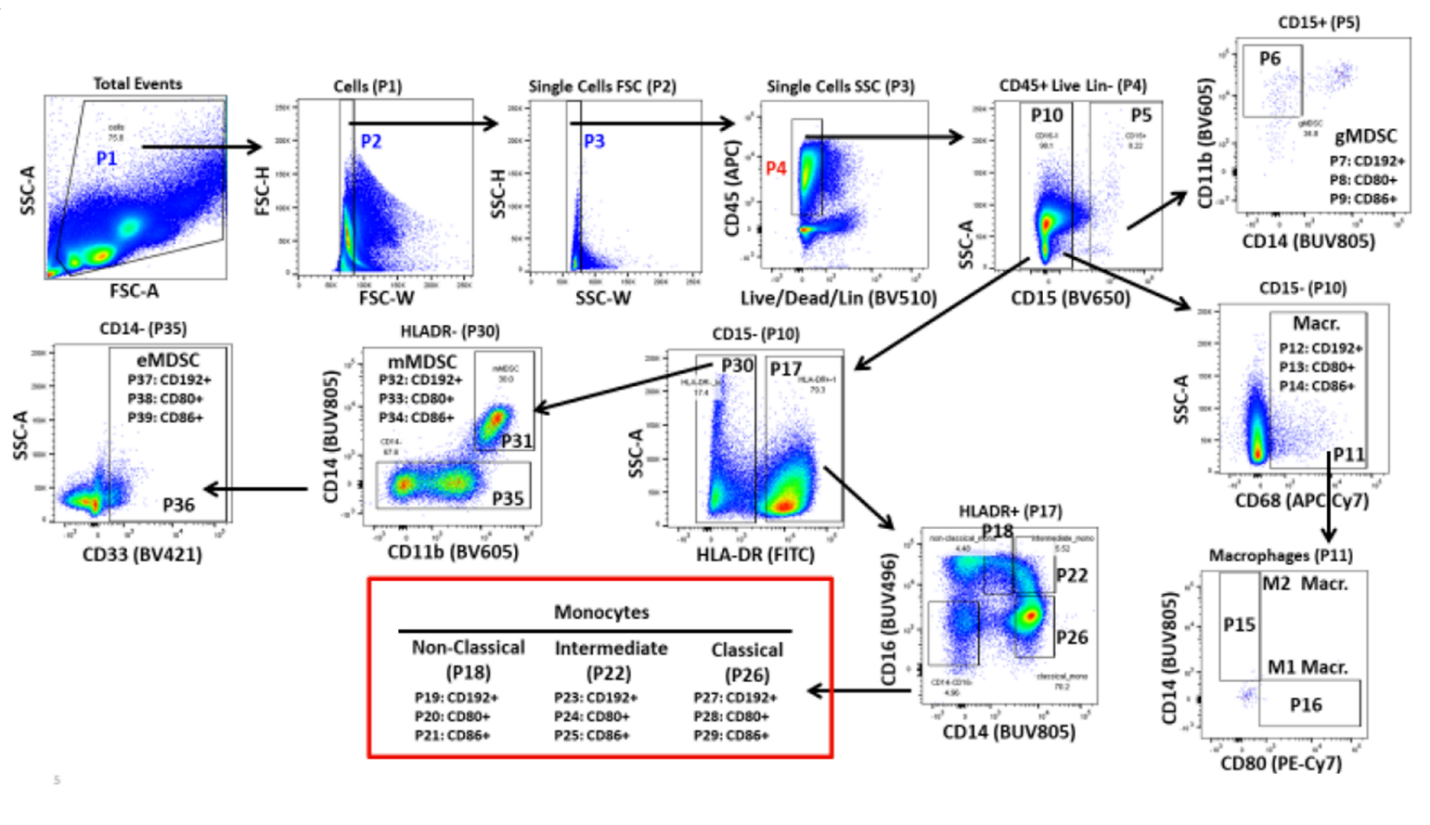
**

Supplement: Supplementary Figure 5 — Gating Strategy for Monocyte Activation in PBMCs [file cir-24-0103_supplementary_figure_5_supps5.docx]

**Supplementary Figure 8.** Gating Strategy for Evaluating T and NK Cell Activation

**
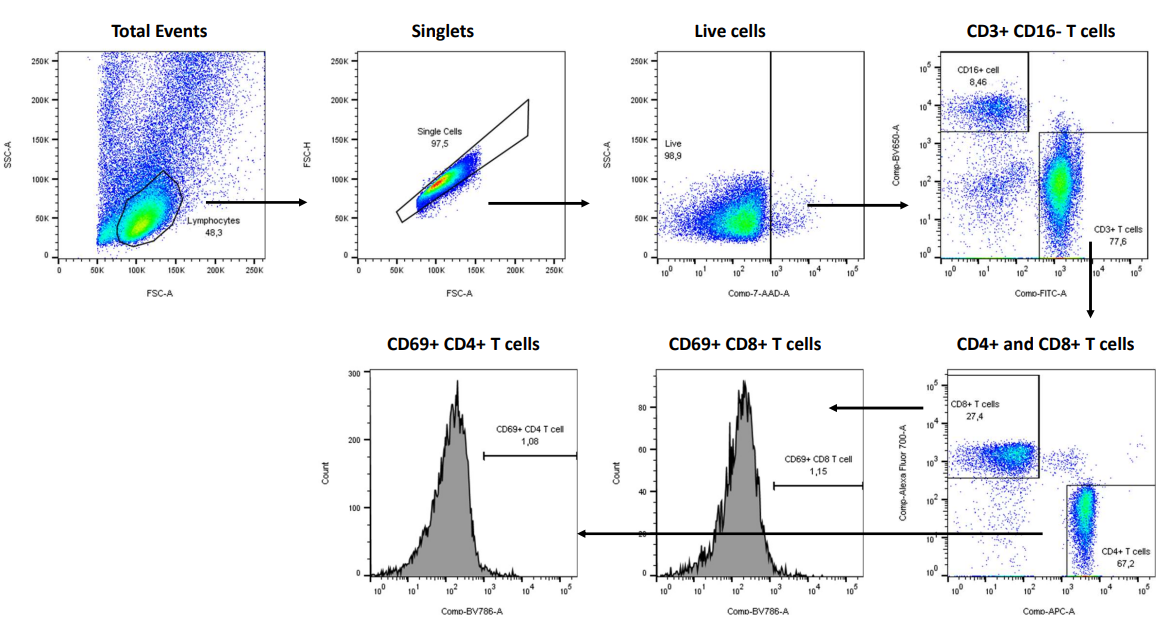
**

**
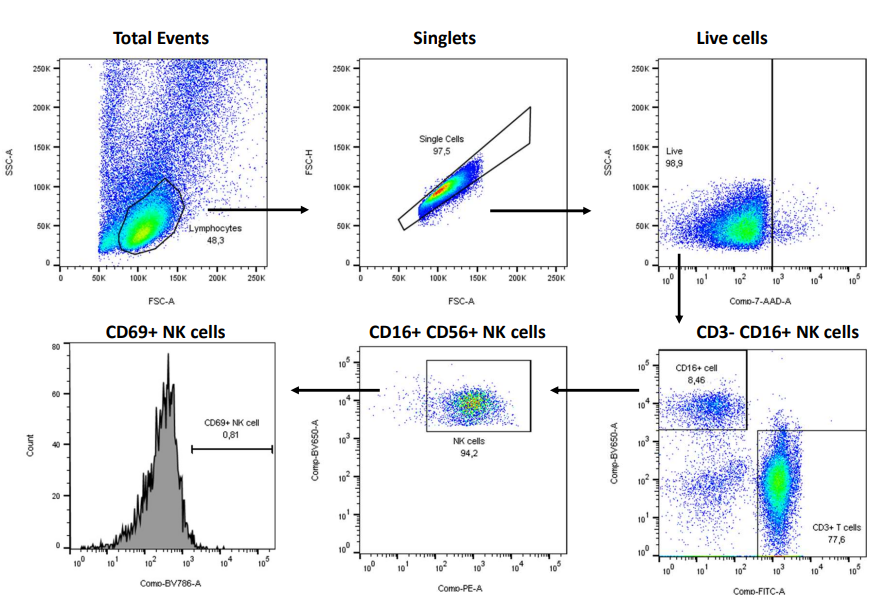
**

Supplement: Supplementary Figure 8 — Gating Strategy for Evaluating T and NK Cell Activation [file cir-24-0103_supplementary_figure_8_supps8.docx]

**Supplementary Figure 9.** Gating Strategy for Murine T Cell Panel

**
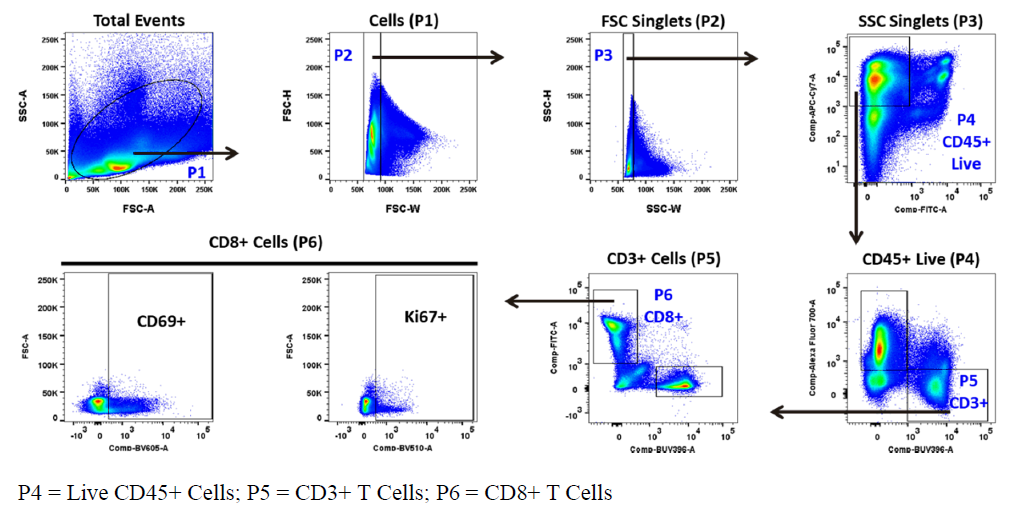
**

Supplement: Supplementary Figure 9 — Gating Strategy for Murine T Cell Panel [file cir-24-0103_supplementary_figure_9_supps9.docx]

**Supplementary Figure 10.** Gating Strategy for Murine Macrophage/Dendritic Cell Panel

**
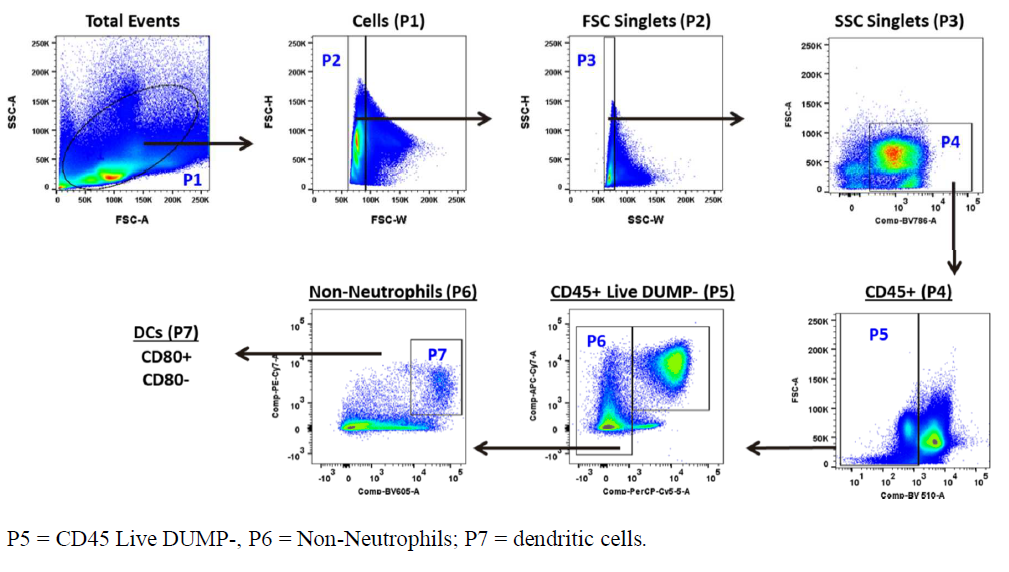
**

Supplement: Supplementary Figure 10 — Gating Strategy for Murine Macrophage/Dendritic Cell Panel [file cir-24-0103_supplementary_figure_10_supps10.docx]

**Supplementary Table 11.** TAK-500 enhances type I IFN response in THP1 cells expressing human CCR2


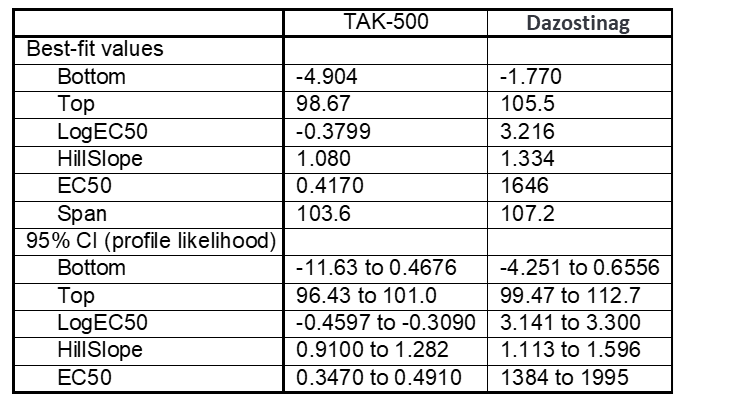

Supplement: Supplementary Table 11 — TAK-500 enhances type I IFN response in THP1 cells expressing human CCR2 [file cir-24-0103_supplementary_table_11_suppst11.docx]

**Supplementary Table 12.** mTAK-500 enhances type I IFN response in THP1 cells expressing murine CCR2


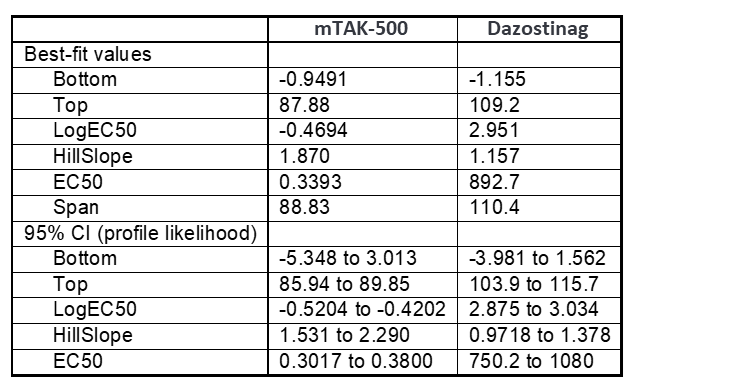

Supplement: Supplementary Table 12 — mTAK-500 enhances type I IFN response in THP1 cells expressing murine CCR2 [file cir-24-0103_supplementary_table_12_suppst12.docx]
