# Supplementary material for: Selective STING Activation in Intratumoral Myeloid Cells via CCR2-Directed Antibody–Drug Conjugate TAK-500
Source: Cancer Immunol Res. 2025 Feb 7;13(5):661–79. doi: 10.1158/2326-6066.CIR-24-0103 (PMC12046323; doi:10.1158/2326-6066.CIR-24-0103)
Supplement: Supplementary Figure 7 — Gating Strategy for Evaluating CCR2 in Dissociated Tumor Cells: Monocyte Panel [file cir-24-0103_supplementary_figure_7_supps7.docx]

**Supplementary Figure 7.** Gating Strategy for Evaluating CCR2 in Dissociated Tumor Cells: Monocyte Panel

**
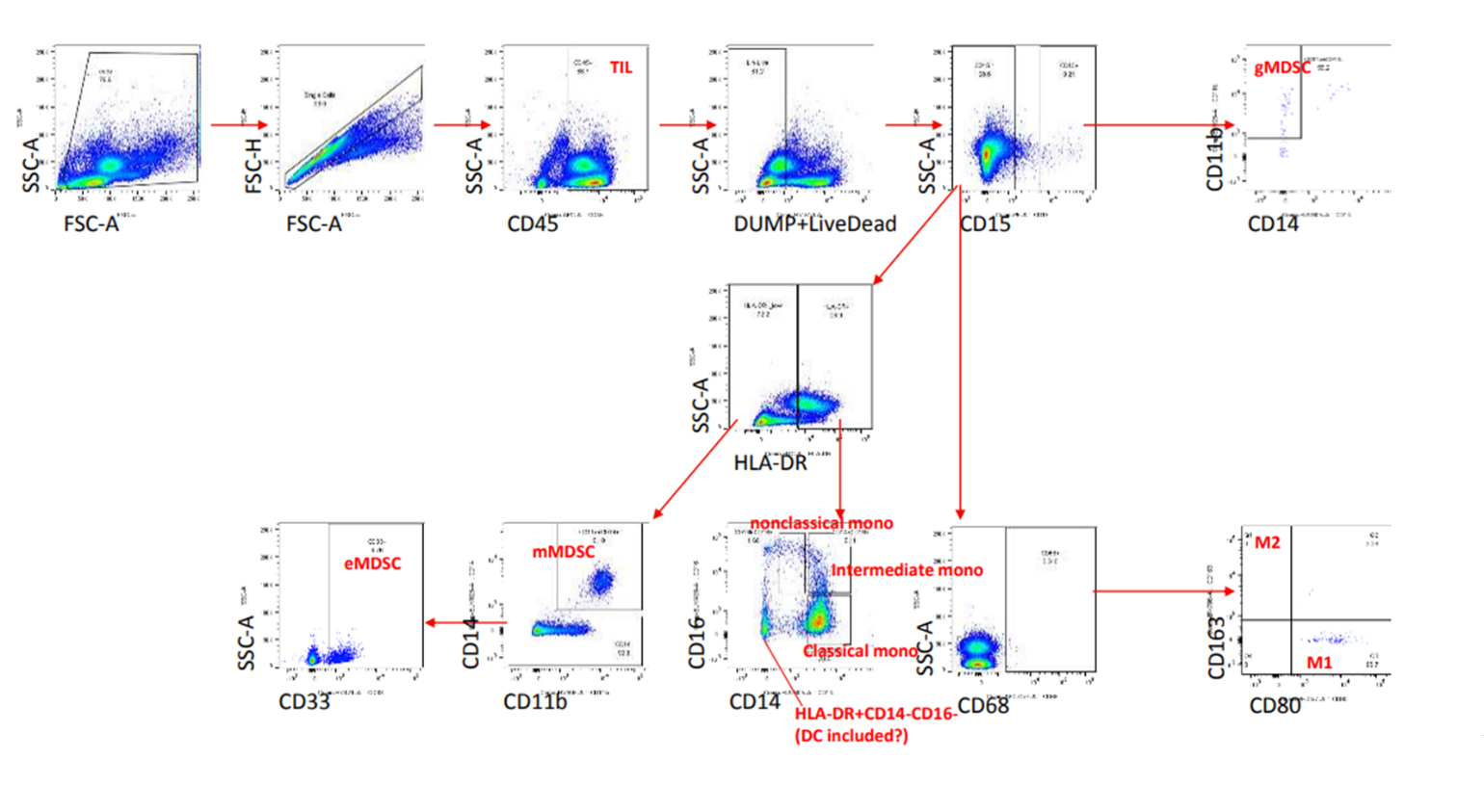
**
