# Supplementary material for: Selective STING Activation in Intratumoral Myeloid Cells via CCR2-Directed Antibody–Drug Conjugate TAK-500
Source: Cancer Immunol Res. 2025 Feb 7;13(5):661–79. doi: 10.1158/2326-6066.CIR-24-0103 (PMC12046323; doi:10.1158/2326-6066.CIR-24-0103)

**Supplementary Figure 11.** Binding Curves Demonstrate Species Specificity for antibody portions of TAK-500 and mTAK-500


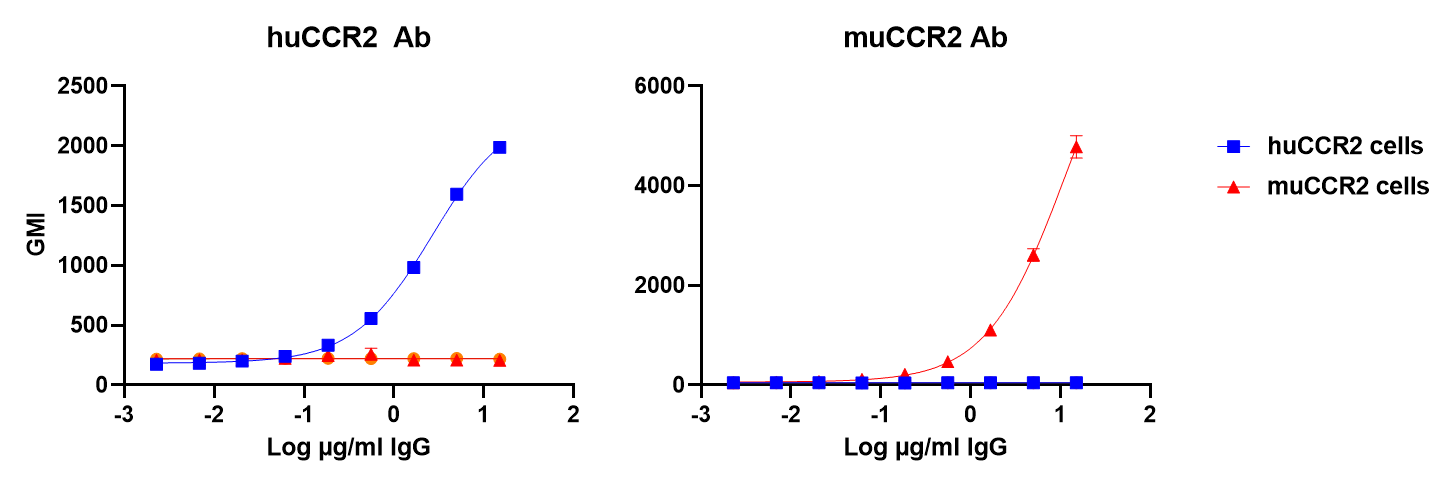

Supplement: Supplementary Figure 11 — Binding Curves Demonstrate Species Specificity for antibody portions of TAK-500 and mTAK-500 [file cir-24-0103_supplementary_figure_11_supps11.docx]
