# Supplementary material for: Selective STING Activation in Intratumoral Myeloid Cells via CCR2-Directed Antibody–Drug Conjugate TAK-500
Source: Cancer Immunol Res. 2025 Feb 7;13(5):661–79. doi: 10.1158/2326-6066.CIR-24-0103 (PMC12046323; doi:10.1158/2326-6066.CIR-24-0103)
Supplement: Supplementary Figure 12 — Complete loss of receptor-mediated TAK-500 and mTAK-500 activity observed on human and murine peripheral blood monocytes lacking cell surface CCR2 expression [file cir-24-0103_supplementary_figure_12_supps12.docx]

**Supplementary Figure 12.** Complete loss of receptor-mediated TAK-500 and mTAK-500 activity observed on human and murine peripheral blood monocytes lacking cell surface CCR2 expression **A.** CRISPR mediated knockout of CCR2 in THP-1 cells confirmed via flow cytometry. **B**. Activation of monocytes as measured by CD86 was evaluated by flow cytometry in THP-1 WT and THP-1 CCR2 KO cells treated with TAK-500. **C**. Reduction in mCCR2 expression on murine monocytes, as induced by MCP-1/CCL2 driven receptor internalization, was confirmed via flow cytometry (Left). Activation of monocytes as measured by CD86 expression was evaluated in murine monocytes with and without CCR2 expression following treatment with mTAK-500 (Right).


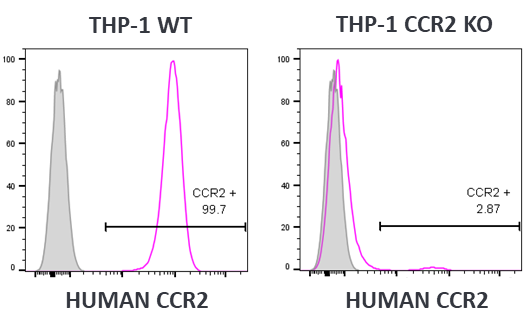
**A.**

**B.**
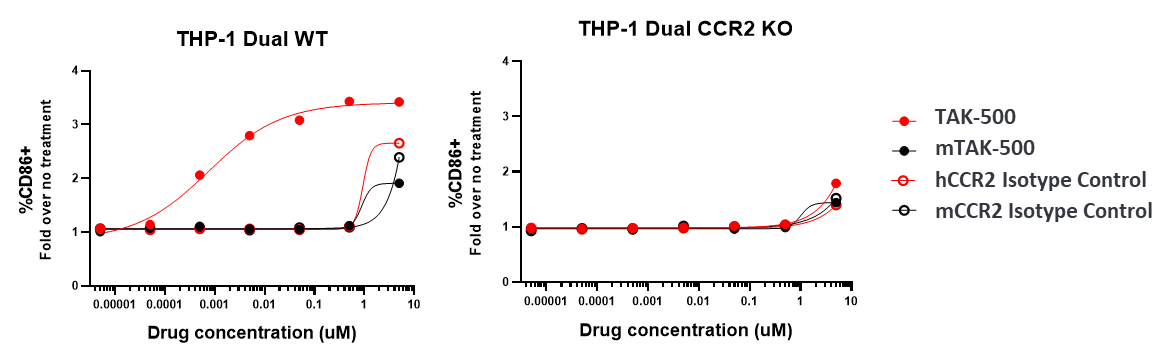


**C.
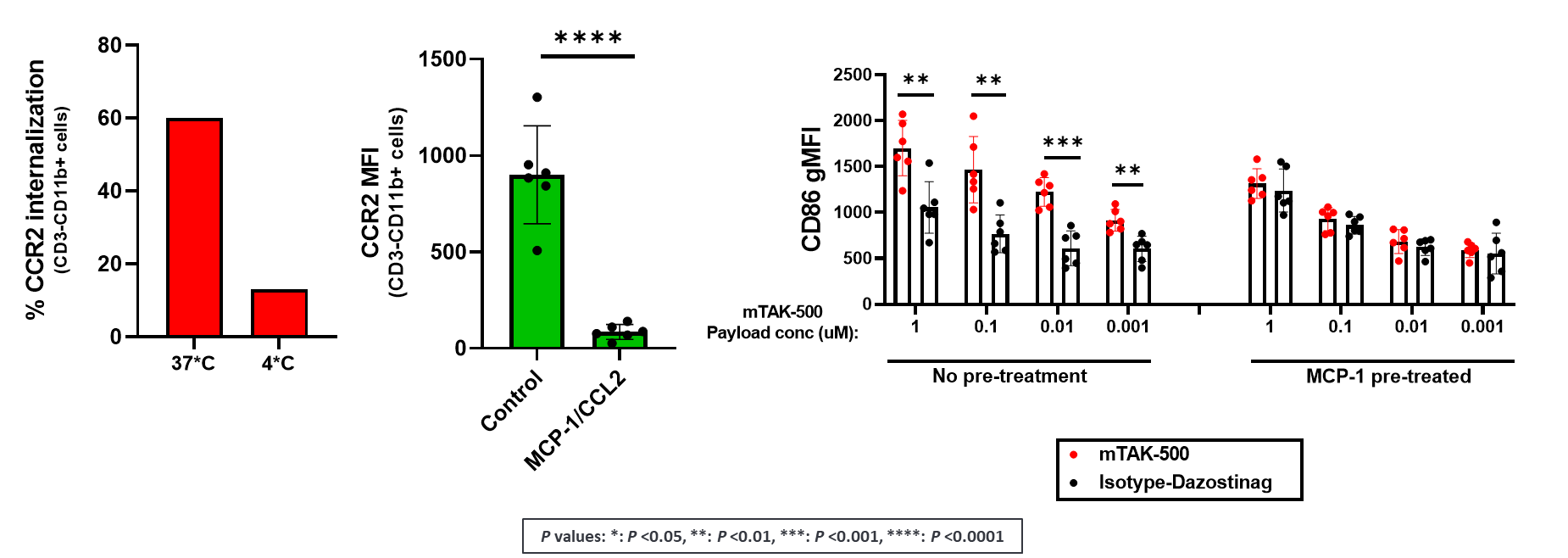
**
