# Supplementary material for: Selective STING Activation in Intratumoral Myeloid Cells via CCR2-Directed Antibody–Drug Conjugate TAK-500
Source: Cancer Immunol Res. 2025 Feb 7;13(5):661–79. doi: 10.1158/2326-6066.CIR-24-0103 (PMC12046323; doi:10.1158/2326-6066.CIR-24-0103)
Supplement: Supplementary Figure 13 — Evaluation of CCR2 expression levels and the impact of TAK-500 treatment on T and NK cell activation and cytokine production in vitro. [file cir-24-0103_supplementary_figure_13_supps13.docx]

**Supplementary Figure 13.** **Evaluation of CCR2 expression levels and the impact of TAK-500 treatment on T and NK cell activation and cytokine production in vitro. A**. Evaluation of CCR2 expression across multiple immune cell subsets using flow cytometry in human breast cancer dissociated tumor cells. **B**. Activation of CD8+ T Cells, CD4+ T Cells, and NK Cells from healthy donor human PBMCs at 24 hours following treatment with TAK-500. **C**. Cytokine induction in healthy donor human PBMCs (left) and isolated NK Cells (Right) following treatment with TAK-500 for 24 hours.

**A.**

**
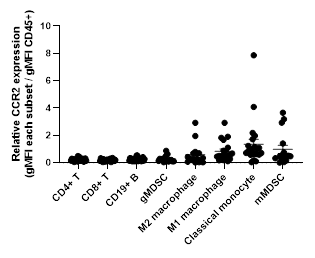
**

**B.**

**
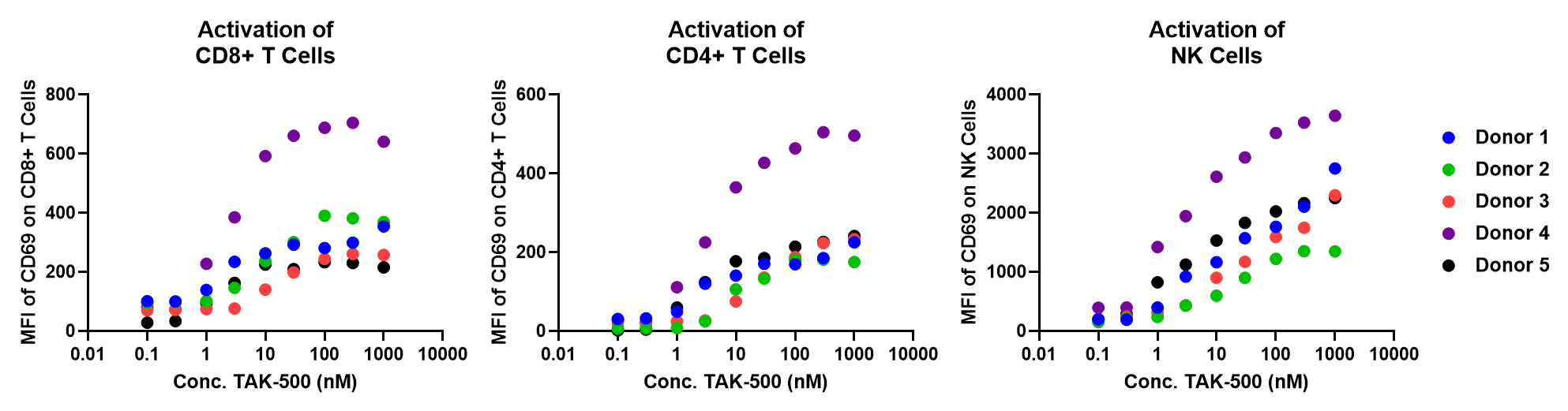
**

**C.**

**
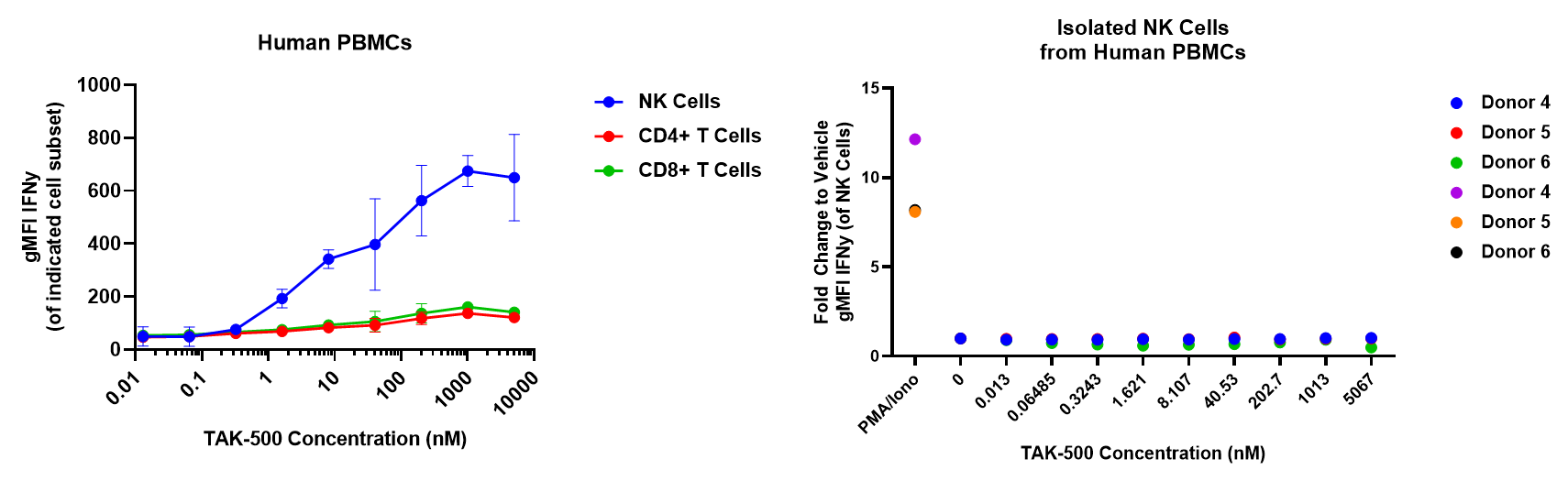
**
