# Supplementary material for: Selective STING Activation in Intratumoral Myeloid Cells via CCR2-Directed Antibody–Drug Conjugate TAK-500
Source: Cancer Immunol Res. 2025 Feb 7;13(5):661–79. doi: 10.1158/2326-6066.CIR-24-0103 (PMC12046323; doi:10.1158/2326-6066.CIR-24-0103)
Supplement: Supplementary Figure 14 — mTAK-500 induced efficacy requires CD8+ T cell mediated adaptive immunity. [file cir-24-0103_supplementary_figure_14_supps14.docx]

**Supplementary Figure 14.** mTAK-500 induced efficacy requires CD8+ T cell mediated adaptive immunity. **A.** Antitumor effect of mTAK-500 in C57BL/6 mice bearing MC38 tumors following depletion of MDSCs **B.** Antitumor effect of mTAK-500 in C57BL/6 mice bearing MC38 tumors following depletion of TAMs **C.** Antitumor effect of mTAK-500 in C57BL/6 (left) and BatF3 knock out (right) mice bearing MC38 tumors to evaluate the impact of loss of cDC1s **D.** Antitumor effect of mTAK-500 in C57BL/6 mice bearing MC38 tumors following depletion of CD4+ T Cells **E.** Antitumor effect of mTAK-500 in C57BL/6 mice bearing MC38 tumors following depletion of CD8+ T Cells.

**A.**   **B.**


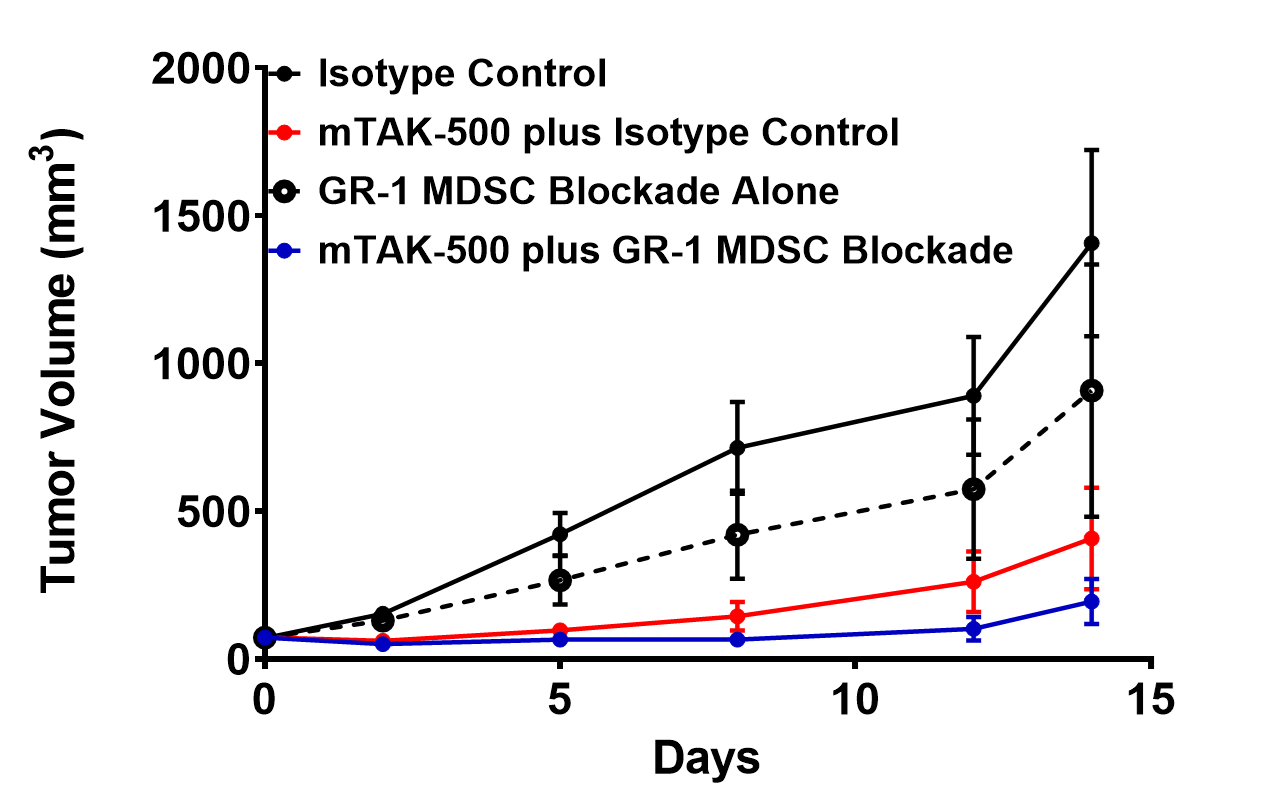

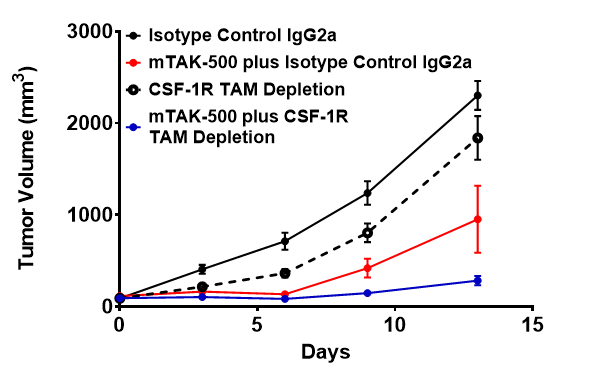


**C.
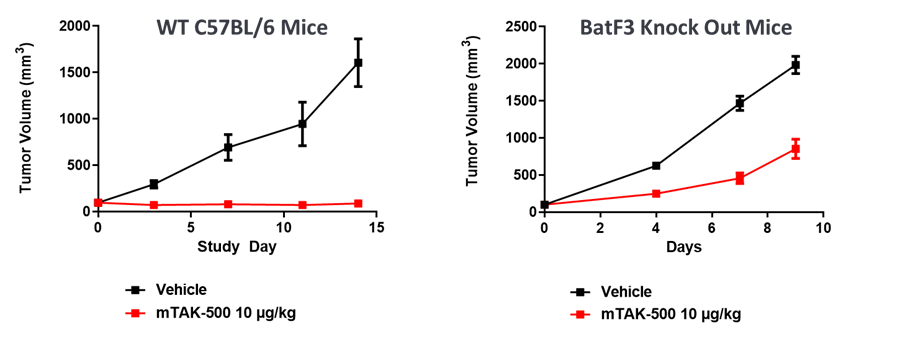
**

**D.**  **E.**
