# Supplementary material for: Selective STING Activation in Intratumoral Myeloid Cells via CCR2-Directed Antibody–Drug Conjugate TAK-500
Source: Cancer Immunol Res. 2025 Feb 7;13(5):661–79. doi: 10.1158/2326-6066.CIR-24-0103 (PMC12046323; doi:10.1158/2326-6066.CIR-24-0103)
Supplement: Supplementary Figure 15 — Evaluation of CCR2 expression in intratumoral mMDSCs within syngeneic tumor bearing mouse models via flow cytometry correlated with the antitumor response of those models to mTAK-500. Models evaluated include C1498, B16F10, MC38, H22, CT26, JC, and Panc02. [file cir-24-0103_supplementary_figure_15_supps15.docx]

**Supplementary Figure 15.** Evaluation of CCR2 expression in intratumoral mMDSCs within syngeneic tumor bearing mouse models via flow cytometry correlated with the antitumor response of those models to mTAK-500. Models evaluated include C1498, B16F10, MC38, H22, CT26, JC, and Panc02.
