# Supplementary material for: Selective STING Activation in Intratumoral Myeloid Cells via CCR2-Directed Antibody–Drug Conjugate TAK-500
Source: Cancer Immunol Res. 2025 Feb 7;13(5):661–79. doi: 10.1158/2326-6066.CIR-24-0103 (PMC12046323; doi:10.1158/2326-6066.CIR-24-0103)
Supplement: Supplementary Table 1 — Cell Lines Utilized Within These Studies [file cir-24-0103_supplementary_table_1_suppst1.docx]

**Supplementary Table 1.** Cell Lines Utilized Within These Studies

| **Cell Line** | **Source** | **Catalog Number** | **Mycoplasma Testing Date** | **Authentication*** |
| --- | --- | --- | --- | --- |
| THP1-Dual™ Human Acute Myeloid Leukemia Cells | Invivogen | thpd-nfis | 6/19/2017 | No |
| CT26.WT Cells | ATCC | CRL-2638 | 6/23/2016 | Yes |
| MC38 Cells | NCI | N/A | 7/20/2020 | Yes |

Abbreviations: ATCC, American Type Culture Collection; N/A, not applicable; NCI, National Cancer Institute.

*Cells were authenticated by IDEXX BioAnalytics CellCheck cell line authentication service with
27-marker short tandem repeat strain analysis.
