# Supplementary material for: Selective STING Activation in Intratumoral Myeloid Cells via CCR2-Directed Antibody–Drug Conjugate TAK-500
Source: Cancer Immunol Res. 2025 Feb 7;13(5):661–79. doi: 10.1158/2326-6066.CIR-24-0103 (PMC12046323; doi:10.1158/2326-6066.CIR-24-0103)
Supplement: Supplementary Table 2 — Example Conditions for generating QTOF- liquid chromatography/mass spectrometry (LCMS) spectra [file cir-24-0103_supplementary_table_2_suppst2.docx]

**Supplementary Table 2.** Example Conditions for generating QTOF- liquid chromatography/mass spectrometry (LCMS) spectra

| Time (min) | Flow (mL/min) | %A | %B |
| --- | --- | --- | --- |
| 0 | 0.35 | 82 | 18 |
| 1 | 0.35 | 82 | 18 |
| 2 | 0.35 | 70 | 30 |
| 19 | 0.5 | 50 | 50 |
| 19.5 | 0.5 | 10 | 90 |
| 21 | 0.5 | 10 | 90 |
| 21.1 | 0.5 | 82 | 18 |
| 22 | 0.5 | 82 | 18 |
