# Supplementary material for: Selective STING Activation in Intratumoral Myeloid Cells via CCR2-Directed Antibody–Drug Conjugate TAK-500
Source: Cancer Immunol Res. 2025 Feb 7;13(5):661–79. doi: 10.1158/2326-6066.CIR-24-0103 (PMC12046323; doi:10.1158/2326-6066.CIR-24-0103)
Supplement: Supplementary Table 3 — Antibodies Used in Murine Receptor Occupancy Flow Panel [file cir-24-0103_supplementary_table_3_suppst3.docx]

**Supplementary Table 3.** Antibodies Used in Murine Receptor Occupancy Flow Panel

| **Conjugate** | **Manufacturer** | **Clone** | **Catalog Number** |
| --- | --- | --- | --- |
| BV510 | BioLegend | 145-2C11 | 100353 |
| BV510 | BioLegend | RA3-6B2 | 103247 |
| BV510 | BioLegend | PK136 | 108738 |
| PerCP-Cy5.5 | BioLegend | M1/70 | 101228 |
| PE-Cy7 | BioLegend | S11 | 143210 |
| APC | BioLegend | N418 | 117310 |
| Super Bright 436 | Thermo Fisher Scientific | HK1.4 | 62-5932-82 |
| PE | BioLegend | SA203G11 | 150610 |
| PE | BioLegend | RMG2a-62 | 407108 |
| PE | Thermo Fisher Scientific | m2a-15F8 | 12-4210-82 |
| PE | R&D Systems | 344701 | F0129 |
| PE | Abcam | N/A | ab74490 |
| N/A | Thermo Fisher Scientific | N/A | L10119 |
