# Supplementary material for: Selective STING Activation in Intratumoral Myeloid Cells via CCR2-Directed Antibody–Drug Conjugate TAK-500
Source: Cancer Immunol Res. 2025 Feb 7;13(5):661–79. doi: 10.1158/2326-6066.CIR-24-0103 (PMC12046323; doi:10.1158/2326-6066.CIR-24-0103)
Supplement: Supplementary Table 4 — Antibodies Used in Human Receptor Occupancy Flow Panel [file cir-24-0103_supplementary_table_4_suppst4.docx]

**Supplementary Table 4.** Antibodies Used in Human Receptor Occupancy Flow Panel

| **Antibody** | **Conjugate** | **Manufacturer** | **Clone** | **Catalog Number** |
| --- | --- | --- | --- | --- |
| CD3 | PE-Cy7 | BD Biosciences | SP34-2 | 557749 |
| CD20 | PE-Cy7 | BD Biosciences | 2H7 | 560735 |
| CD56 | PE-Cy7 | BioLegend | 5.1H11 | 362510 |
| HLA-DR | FITC | BioLegend | L243 | 307604 |
| CD192 | PE | BioLegend | K036C2 | 357206 |
| CD16 | APC | BioLegend | 3G8 | 302012 |
| Cd159a | PE-Cy7 | Beckman Coulter | Z199 | B10246 |
| Mouse Anti-Human IgG | PE | Southern Biotech | JDC-10 | 9040-09 |
| CD14 | BV510 | BioLegend | M5E2 | 301842 |
| Live Dead Fixable Near IR | N/A | Thermo Fisher Scientific | N/A | L10119 |
