# Supplementary material for: Selective STING Activation in Intratumoral Myeloid Cells via CCR2-Directed Antibody–Drug Conjugate TAK-500
Source: Cancer Immunol Res. 2025 Feb 7;13(5):661–79. doi: 10.1158/2326-6066.CIR-24-0103 (PMC12046323; doi:10.1158/2326-6066.CIR-24-0103)
Supplement: Supplementary Table 5 — Antibodies Used in Monocyte Activation Flow Panel [file cir-24-0103_supplementary_table_5_suppst5.docx]

**Supplementary Table 5.** Antibodies Used in Monocyte Activation Flow Panel

| Antibody | Conjugate | Manufacturer | City | State | Country | Clone | Catalog No. | Dilution |
| --- | --- | --- | --- | --- | --- | --- | --- | --- |
| CD14 | BUV805 | Becton, Dickinson and Company | Franklin Lakes | NJ | USA | MSE2 | 565779 | 1 to 200 |
| CD16 | BUV496 | Becton, Dickinson and Company | Franklin Lakes | NJ | USA | 3G8 | 564653 | 1 to 200 |
| CD192 | PerCP- Cy5.5 | BioLegend, Inc. | San Diego | CA | USA | K036C2 | 357204 | 1 to 100 |
| CD11b | BV 605 | BioLegend, Inc. | San Diego | CA | USA | M1/70 | 101257 | 1 to 100 |
| CD33 | BV 421 | BioLegend, Inc. | San Diego | CA | USA | WM53 | 303416 | 1 to 100 |
| HLA-DR | FITC | BioLegend, Inc. | San Diego | CA | USA | L243 | 980402 | 1 to 100 |
| CD86 | BV 785 | BioLegend, Inc. | San Diego | CA | USA | IT2.2 | 305442 | 1 to 100 |
| CD80 | PE- Cy7 | BioLegend, Inc. | San Diego | CA | USA | 2D10 | 305218 | 1 to 100 |
| Ghost Dye Violet 510 | Ghost Dye Violet 510 | Tonbo Bio | San Diego | CA |  | N/A | 13-0870-T100 | 1 to 1000 |
| CD15 | BV 650 | BioLegend, Inc. | San Diego | CA | USA | W6D3 | 323034 | 1 to 100 |
| CD45 | APC | BioLegend, Inc. | San Diego | CA | USA | HI30 | 304037 | 1 to 100 |
| CD68 | APC-Cy7 | BioLegend, Inc. | San Diego | CA | USA | Y1/82A | 333822 | 1 to 100 |
