# Supplementary material for: Selective STING Activation in Intratumoral Myeloid Cells via CCR2-Directed Antibody–Drug Conjugate TAK-500
Source: Cancer Immunol Res. 2025 Feb 7;13(5):661–79. doi: 10.1158/2326-6066.CIR-24-0103 (PMC12046323; doi:10.1158/2326-6066.CIR-24-0103)
Supplement: Supplementary Table 6 — T Cell Panel for Evaluating Dissociated Tumor Cells [file cir-24-0103_supplementary_table_6_suppst6.docx]

**Supplementary Table 6.** T Cell Panel for Evaluating Dissociated Tumor Cells

| **Antibody** | **Conjugate** | **Manufacturer** | **Clone** | **Catalog Number** | **Dilution** |
| --- | --- | --- | --- | --- | --- |
| CD45 | PerCP-Cy5.5 | BioLegend | HI30 | 304028 | 1:100 |
| CD34 | BV510 | BioLegend | 581 | 343528 | 1:100 |
| CD45RA | PE-Cy7 | BioLegend | HI100 | 304126 | 1:100 |
| CD19 | BUV395 | BD Biosciences | SJ25C1 | 563549 | 1:100 |
| CD20 | BV421 | BioLegend | 2H7 | 302330 | 1:100 |
| IgD | BV605 | BioLegend | IA6-2 | 348232 | 1:100 |
| CD27 | BV711 | BioLegend | O323 | 302834 | 1:100 |
| CD3 | PE/Dazzle 594 | BioLegend | UCHT1 | 300450 | 1:100 |
| CD4 | BUV805 | BD Biosciences | SK3 | 564910 | 1:100 |
| CD8 | BUV737 | BD Biosciences | SK1 | 564629 | 1:100 |
| CD25 | PE | BioLegend | M-A251 | 356104 | 1:100 |
| FoxP3 | Alexa Fluor 488 | BioLegend | 206D | 320112 | 1:100 |
| CCR7 | Super Bright 780 | Thermo Fisher Scientific | 3D12 | 78-1979-42 | 1:20 |
| CD192 | APC | BioLegend | K036C2 | 357208 | 1:100 |
| Live Dead Near IR | N/A | Thermo Fisher Scientific | N/A | L10119 | N/A |
| CD11b | APC-Cy7 | BioLegend | M1/70 | 101226 | 1:50 |
| CD14 | APC-Cy7 | BioLegend | M5E2 | 301820 | 1:50 |
| CD56 | APC-Cy7 | BioLegend | HCD56 | 318332 | 1:50 |
