# Supplementary material for: Selective STING Activation in Intratumoral Myeloid Cells via CCR2-Directed Antibody–Drug Conjugate TAK-500
Source: Cancer Immunol Res. 2025 Feb 7;13(5):661–79. doi: 10.1158/2326-6066.CIR-24-0103 (PMC12046323; doi:10.1158/2326-6066.CIR-24-0103)
Supplement: Supplementary Table 7 — Monocyte Panel for Evaluating Dissociated Tumor Cells [file cir-24-0103_supplementary_table_7_suppst7.docx]

**Supplementary Table 7.** Monocyte Panel for Evaluating Dissociated Tumor Cells

| **Antibody** | **Conjugate** | **Manufacturer** | **Clone** | **Catalog Number** | **Dilution** |
| --- | --- | --- | --- | --- | --- |
| CD14 | BUV805 | BD Biosciences | M5E2 | 565779 | 1:100 |
| CD16 | BUV496 | BD Biosciences | 3G8 | 564654 | 1:100 |
| CD192 | PerCP-Cy5.5 | BioLegend | K036C2 | 357204 | 1:100 |
| CD11b | BV605 | BioLegend | M1/70 | 101257 | 1:100 |
| CD33 | BV421 | BioLegend | WM53 | 303416 | 1:100 |
| HLA-DR | FITC | BioLegend | L243 | 980402 | 1:100 |
| CD163 | BV785 | BioLegend | GHI/61 | 333631 | 1:100 |
| CD80 | PE-Cy7 | BioLegend | 2D10 | 305218 | 1:100 |
| CD15 | PE | BioLegend | HI98 | 301906 | 1:100 |
| CD45 | APC | BioLegend | 433H | 304037 | 1:100 |
| CD68 | APC-Cy7 | BioLegend | Y1/82A | 333822 | 1:100 |
| Ghost510 | BV510 | Cell Signalling Technology | N/A | 59863 | N/A |
| CD3 | BV510 | BD Biosciences | UCHT-1 | 563109 | 1:50 |
| CD19 | BV510 | BD Biosciences | SJ25C1 | 562947 | 1:50 |
| CD56 | BV510 | BD Biosciences | NCAM16.2 | 563041 | 1:50 |
