# Supplementary material for: Selective STING Activation in Intratumoral Myeloid Cells via CCR2-Directed Antibody–Drug Conjugate TAK-500
Source: Cancer Immunol Res. 2025 Feb 7;13(5):661–79. doi: 10.1158/2326-6066.CIR-24-0103 (PMC12046323; doi:10.1158/2326-6066.CIR-24-0103)
Supplement: Supplementary Table 8 — Flow Panel for T and NK Cell Activation in Human PBMCs [file cir-24-0103_supplementary_table_8_suppst8.docx]

**Supplementary Table 8.** Flow Panel for T and NK Cell Activation in Human PBMCs

| **Antibody** | **Conjugate** | **Manufacturer** | **Clone** | **Catalog Number** | **Dilution** |
| --- | --- | --- | --- | --- | --- |
| CD3 | FITC | BD Biosciences | UCHT1 | 561807 | 1:20 |
| CD69 | BV786 | BD Biosciences | FN50 | 563834 | 1:20 |
| CD8 | Alexa 700 | BioLegend | SK1 | 344724 | 1:20 |
| CD16 | BV650 | BioLegend | 3G8 | 302042 | 1:20 |
| CD4 | APC | BD Biosciences | RPA-T4 | 561841 | 1:20 |
| CD56 | PE | Miltenyi Biotec | REA196 | 130-113-312 | 1:50 |
| 7-AAD | PE Cy5 | Thermo Fisher Scientific | N/A | A1310 | N/A |
