# Supplementary material for: Selective STING Activation in Intratumoral Myeloid Cells via CCR2-Directed Antibody–Drug Conjugate TAK-500
Source: Cancer Immunol Res. 2025 Feb 7;13(5):661–79. doi: 10.1158/2326-6066.CIR-24-0103 (PMC12046323; doi:10.1158/2326-6066.CIR-24-0103)
Supplement: Supplementary Table 9 — Antibodies Used in T Cell Panel [file cir-24-0103_supplementary_table_9_suppst9.docx]

**Supplementary Table 9.** Antibodies Used in T Cell Panel

| Antibody | Conjugate | Manufacturer | City | State | Country | Clone | Catalog No. | Dilution |
| --- | --- | --- | --- | --- | --- | --- | --- | --- |
| CD192 | Brilliant Violet 421 | BioLegend | San Diego | CA | USA | SA203G11 | 150605 | 1:50 |
| CD45 | APC-Cy7 | BioLegend | San Diego | CA | USA | 30-F11 | 103116 | 1:100 |
| CD3 | PerCP-Cy5.5 | BioLegend | San Diego | CA | USA | 17A2 | 100218 | 1:100 |
| CD4 | BUV395 | BD Biosciences | Franklin Lakes | NJ | USA | GK1.5 | 563790 | 1:100 |
| CD8 | BUV737 | BD Biosciences | Franklin Lakes | NJ | USA | 53-6.7 | 564297 | 1:400 |
| Ki-67 | Brilliant Violet 510 | BD Biosciences | Franklin Lakes | NJ | USA | B56 | 563462 | 1:100 |
| ICOS | PE-Cy7 | BioLegend | San Diego | CA | USA | C398.4A | 313520 | 1:200 |
| CD44 | Brilliant Violet 786 | BD Biosciences | Franklin Lakes | NJ | USA | IM7 | 563736 | 1:100 |
| CD62L | PE-CF594 | BD Biosciences | Franklin Lakes | NJ | USA | MEL-14 | 562404 | 1:100 |
| CD25 | Brilliant Violet 650 | BioLegend | San Diego | CA | USA | PC61 | 102038 | 1:100 |
| CD45R | Alexa Fluor 700 | BioLegend | San Diego | CA | USA | RA3-6B2 | 103232 | 1:200 |
| CD69 | Brilliant Violet 605 | BD Biosciences | Franklin Lakes | NJ | USA | H1.2F3 | 563290 | 1:100 |
| NKp46 | Brilliant Violet 711 | BioLegend | San Diego | CA | USA | 29A1.4 | 137621 | 1:100 |
| Live/Dead Fix Green | Live/Dead Fix Green | Thermo Fisher | Waltham | MA | USA | N/A | L23101 | 1:1000 |
| AH1 Tetramer | PE | Novus Biologicals | Centennial | CO | USA | AH1 | NBP1-05209PE | 1:100 |
