# Supplementary material for: Selective STING Activation in Intratumoral Myeloid Cells via CCR2-Directed Antibody–Drug Conjugate TAK-500
Source: Cancer Immunol Res. 2025 Feb 7;13(5):661–79. doi: 10.1158/2326-6066.CIR-24-0103 (PMC12046323; doi:10.1158/2326-6066.CIR-24-0103)
Supplement: Supplementary Table 10 — Antibodies Used in Macrophage/Dendritic Cell Panel [file cir-24-0103_supplementary_table_10_suppst10.docx]

**Supplementary Table 10.** Antibodies Used in Macrophage/Dendritic Cell Panel

| Antibody | Conjugate | Manufacturer | City | State | Country | Clone | Catalog No. | Dilution |
| --- | --- | --- | --- | --- | --- | --- | --- | --- |
| iNOS | BUV805 | ebioscience | San Diego | CA | USA | CXNFT | 61-5920-82 | 1:100 |
| Arginase 1 | BUV496 | Thermo Fisher | Waltham | MA | USA | A1exF5 | 53-3697-82 | 1:100 |
| CD45 | PerCP- Cy5.5 | BD Biosciences | Franklin Lakes | NJ | USA | 30-F11 | 564225 | 1:200 |
| F4/80 | BV 605 | BD Biosciences | Franklin Lakes | NJ | USA | T45-2342 | 565614 | 1:200 |
| MHC II | BV 421 | BD Biosciences | Franklin Lakes | NJ | USA | M5/114.15.2 | 563413 | 1:300 |
| CD11b | FITC | BD Biosciences | Franklin Lakes | NJ | USA | M1/70 | 564443 | 1:100 |
| CD11c | BV 785 | BD Biosciences | Franklin Lakes | NJ | USA | HL3 | 558079 | 1:200 |
| Ly-6C | PE- Cy7 | BioLegend | San Diego | CA | USA | HK1.4 | 128026 | 1:200 |
| Ly-6G | Ghost Dye Violet 510 | BioLegend | San Diego | CA | USA | 1A8 | 127616 | 1:200 |
| CD86 | BV 650 | BD Biosciences | Franklin Lakes | NJ | USA | GL1 | 565479 | 1:100 |
| CD80 | APC | BioLegend | San Diego | CA | USA | 16-10A1 | 104732 | 1:100 |
| Mannose Receptor | APC-Cy7 | ebioscience | San Diego | CA | USA | MR6F3 | 12-2061-82 | 1:50 |
| CD115 | Brilliant Violet 711 | BioLegend | San Diego | CA | USA | AFS98 | 135515 | 1:100 |
| CD192 | Brilliant Violet 421 | BioLegend | San Diego | CA | USA | SA203G11 | 150605 | 1:200 |
| Live/Dead Fix Aqua | Live/Dead Fix Aqua | Thermo Fisher | Waltham | MA | USA | N/A | L34957 | 1:1000 |
| CD45R | Brilliant Violet 510 | Biolegend | San Diego | CA | USA | RA3-6B2 | 103248 | 1:200 |
| CD3 | Brilliant Violet 510 | Biolegend | San Diego | CA | USA | 17A2 | 100535 | 1:100 |
| NKp46 | Brilliant Violet 510 | Biolegend | San Diego | CA | USA | 29A1.4 | 137023 | 1:100 |
| TER-119 | Brilliant Violet 510 | Biolegend | San Diego | CA | USA | TER-119 | 116237 | 1:100 |
