# Supplementary material for: Selective STING Activation in Intratumoral Myeloid Cells via CCR2-Directed Antibody–Drug Conjugate TAK-500
Source: Cancer Immunol Res. 2025 Feb 7;13(5):661–79. doi: 10.1158/2326-6066.CIR-24-0103 (PMC12046323; doi:10.1158/2326-6066.CIR-24-0103)
Supplement: Supplementary Table 13 — Pharmacokinetic Parameters of Total Ab, Conjugated Dazostinag, and Deconjugated Dazostinag in Female C57BL/6 Mice Bearing MC38 Tumors After Intravenous Administration of mTAK-500 at 2, 10, and 50 µg/kg [file cir-24-0103_supplementary_table_13_suppst13.docx]

**Supplementary Table 13.** Pharmacokinetic Parameters of Total Ab, Conjugated Dazostinag, and Deconjugated Dazostinag in Female C57BL/6 Mice Bearing MC38 Tumors After Intravenous Administration of mTAK-500 at 2, 10, and 50 µg/kg

Ab: antibody; AUC_all_: area under the concentration-time curve from the start of dose administration to the time of the last observation; AUC_0-24h_,: area under the concentration-time curve from time 0 to 24 hours; AUC_∞_: AUC from time 0 to infinity, calculated using the observed value of the last quantifiable concentration; Cl.: clearance; NA: not applicable; NC: not calculated; NR: not reported, due to the %AUC_extrapolated_ (percentage of the AUC extrapolated to infinity observed from time of the last observation [t_last_] to infinity) represented more than 20% of the total area; Vss: volume of distribution at steady state.

Pharmacokinetic parameters for deconjugated dazostinag (TAK-676) at 2 and 10 µg/kg were not reported due to all values below quantitation limit. Molecular weight of dazostinag (TAK-676) is 710.52 g/mol. Conversion factor I ng/mL = 1.41 nM



Molecular weight of TAK-500 is 156,000 g/mol. Conversion factor 1 µg/mL = 6.41 nM

^a^AUC_0-24h_ was used for exposure comparison between different dosing groups.

^b^The antibody-drug conjugate based dose was used for Total Ab (TAb). Concentration of TAb is based on antibody.

^c^Payload based dose was used for conjugated dazostinag (TAK-676) and deconjugated dazostinag (TAK-676).
