# Supplementary material for: Selective STING Activation in Intratumoral Myeloid Cells via CCR2-Directed Antibody–Drug Conjugate TAK-500
Source: Cancer Immunol Res. 2025 Feb 7;13(5):661–79. doi: 10.1158/2326-6066.CIR-24-0103 (PMC12046323; doi:10.1158/2326-6066.CIR-24-0103)
Supplement: Supplementary Table 14 — Clinicopathologic characteristics of NSCLC, CRC and PDAC cohorts [file cir-24-0103_supplementary_table_14_suppst14.docx]

**Supplementary Table 14.** Clinicopathologic characteristics of NSCLC, CRC and PDAC cohorts

|  | **NSCLC Cohort 1 (n=172)** | **NSCLC Cohort 2 (n=239)** | **NSCLC**  **Cohort 3 (n=119)** | **CRC Cohort 1 (n=217)** | **CRC Cohort 2 (n=133)** | **PDAC Cohort (n=228)** |
| --- | --- | --- | --- | --- | --- | --- |
|  |  |  |  |  |  |  |
| **Median age (range)** | 65 (33-89) | 67 (37-91) | n/a | 72 (30-97) | 69 (28-91) | 69 (34-87) |
|  |  |  |  |  |  |  |
| **Gender** |  |  |  |  |  |  |
| Male | 82 (48%) | 100 (42%) | 40 (34%) | 98 (45%) | 62 (47%) | 111 (49%) |
| Female | 89 (52%) | 118 (49%) | 79 (66%) | 116 (53%) | 59 (44%) | 117 (51%) |
| **Stage** |  |  |  |  |  |  |
| Stage I-II | 121 (70%) | 173 (72%) | n/a | 89 (41%) | 81 (61%) | 210 (92%) |
| Stage III-IV | 49 (28%) | 38 (16%) |  | 112 (52%) | 52 (39%) | 8 (4%) |
| **Histology** |  |  |  |  |  |  |
| LUAD | 97 (56%) | 113 (47%) | 119 (100%) |  |  |  |
| LUSC | 30 (17%) | 58 (24%) | 0 | n/a | n/a | n/a |
| Others | 33 (19%) | 44 (18%) | 0 |  |  |  |
| **Smoking status** |  |  |  |  |  |  |
| Non/former smoker | 12 (7%) | 145 (61%) | n/a | 164 (76%) | n/a | 192 (84%) |
| Smoker | 158 (92%) | 58 (24%) |  | 23 (11%) |  | 25 (11%) |
| **Mutation in** |  |  |  |  |  |  |
| *EGFR* | n/a | n/a | 26 (22%) |  |  |  |
| *KRAS*- |  |  | 38 (32%) | n/a | n/a | n/a |
| *EGFR/KRAS*-wt |  |  | 55 (46%) |  |  |  |
| **Microsatellite stability** |  |  |  |  |  |  |
| MSI-H | n/a | n/a | n/a | 19 (9%) | 31 (23%) | n/a |
| MSS |  |  |  | 166 (76%) | 102 (77%) |  |
